# Zn(II)-Responsive Peptide Hydrogels with Tunable Mechanical Properties

**DOI:** 10.1021/acsomega.5c11025

**Published:** 2026-02-13

**Authors:** Alexia Tialiou, Christopher J. Serpell, Çağrı Özsan, Lingcong Ge, Angelo Frei, Jia Min Chin, Bernhard K. Keppler, Michael R. Reithofer

**Affiliations:** † Institute of Inorganic Chemistry, Faculty of Chemistry, University of Vienna, Währinger Str. 42, 1090 Vienna, Austria; ‡ Vienna Doctoral School in Chemistry (DoSChem), University of Vienna, Währinger Str. 42, 1090 Vienna, Austria; § School of Pharmacy, 4919 University College London, 29/39 Brunswick square, London WC1N1AX, U.K.; ∥ Department of Chemistry, University of York, Heslington, York YO10 5DD, U.K.; ⊥ Institute of Functional Materials and Catalysis, Faculty of Chemistry, University of Vienna, Währinger Str. 42, 1090 Vienna, Austria; # Research Cluster “Translational Cancer Therapy Research”, University of Vienna and Medical University of Vienna, Währinger Str. 42, 1090 Vienna, Austria

## Abstract

Metal-coordinated peptide assemblies represent a versatile platform for functional biomaterials; here we describe Zn­(II)-driven hydrogelation of short amphiphilic peptides. To this end, we synthesized two short amphiphilic hexapeptides, Ac-LIVKHH-NH_2_ and Fmoc-LIVKHH-NH_2_, using standard Fmoc/Boc solid-phase peptide synthesis. Upon interaction with Zn­(II) salts in aqueous solution (pH 7), these peptides encapsulate large volumes of water to form metallo-hydrogels. The Zn­(II)-mediated gelation and structural organization of the resulting supramolecular architectures were examined using circular dichroism (CD), Fourier transform infrared spectroscopy (FTIR), transmission electron microscopy (TEM) and scanning electron microscopy (SEM), respectively. Oscillatory rheology and thixotropy measurements confirmed the viscoelastic and shear-recoverable properties of the hydrogels. Zn­(II) coordination was found to play a key role in enhancing mechanical robustness, while the thixotropic behavior highlights their potential as injectable carriers and bioinks for 3D printing. Antibacterial assays against *Escherichia coli* and *Staphylococcus aureus* further revealed moderate inhibition zones, indicating additional functional utility. Overall, this work provides new insights into the Zn­(II)-responsive assembly of short amphiphilic peptides and establishes a foundation for their development in biomaterials and materials science.

## Graphical abstract

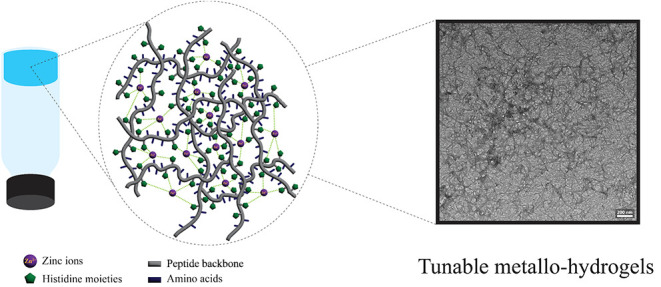

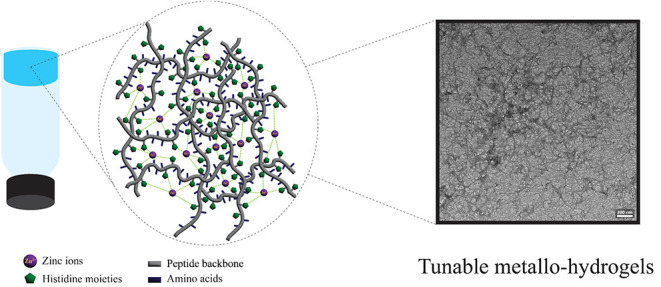

## 1 Introduction

Molecular self-assembly refers to the spontaneous and reversible organization of molecules into higher-order structures.^1,2^ This phenomenon is highly prevalent in nature, where assembled entities such as DNA or proteins exhibit distinctive biological functions.^3^ Peptide-based supramolecular architectures stand out for their biocompatibility, biodegradability, and ease of synthesis.^4,5^ Such structures are based on the organization of peptides into secondary structures such as β-sheet or α-helix, which can then undergo hierarchical and multiscale assembly processes.^6–8^ These structures rely on noncovalent interactions, such as hydrogen bonds, hydrophobic interactions, van der Waals forces, electrostatic- and π–π interactions, respectively.^9,10^

In aqueous solution, these self-assembled peptide architectures can also yield hydrogels by entrapping large amounts of water.^11–13^ To generate peptide hydrogels, the use of short peptides is particularly appealing as it offers robustness, scalability, and cost-effectiveness.^4,8,14–16^ Besides the rational design of short peptides, a common strategy to modulate the self-assembling properties involves protection of the *N*- and/or *C*-terminus through the use of acetyl or large aromatic groups such as fluorenylmethoxycarbonyl protecting group (Fmoc).^15,17–19^ This modification reduces charge repulsions and introduces π-stacking/hydrophobic contributions, thereby promoting self-assembly and hydrogel formation.^20–22^

Metal ions are frequently employed to trigger peptide assembly, thereby leading to hydrogelation.^23–26^ Such metal ion-coordinated peptide hydrogels hold significant potential, as they exploit the unique physicochemical characteristics of metals, such as Zn­(II), which play essential roles in biological processes and disease-related protein assemblies.^14,27–30^ For instance, zinc, known for its antimicrobial and bacteriostatic properties, is beneficial in wound healing and infection prevention applications.^23,31–35^ Additionally, zinc plays a crucial role in collagen synthesis, imparting tensile strength to the newly formed tissue at the wound site.^36^ However, a comprehensive understanding of the impact of metal coordination on peptide assembly processes affording hydrogels remains limited. The addition of metal ions through metal–ligand coordination offers a method to tune the mechanical stiffness of hydrogels, complementing the other approaches of manipulating cross-link density, peptide concentration, or pH, which can influence the essential biological functions of such gels.^24,37–40^ Metal salts or complexes have been utilized to induce gelation in peptide solutions, enabling formation of stable gels through ordered aggregates formed by metal-peptide interactions.^12,25,29,37,38,40^ For example, Mishra and co-workers investigated the influence of metal salts on the self-assembling behavior of Ac-LIVAGD and Ac-IVD, revealing how cation coordination and ionic strength affect hydrogelation, facilitating controlled modulation of the mechanical properties of Ac-LIVAGD hydrogel for targeted biomedical applications.^41^

Herein, short amphiphilic peptides were developed with specific binding affinity for biologically benign metal salts, particularly zinc. In aqueous conditions, the interactions between the histidine moieties of peptides and Zn­(II) resulted in the formation of coordination polymers incorporating zinc as a cross-linker, ultimately leading to metallo-hydrogel formation. We studied the zinc-responsive behavior of the metallo-hydrogels, and the resulting impact on their mechanical properties, viscoelastic character, and recovery capabilities. The observed effects demonstrate how zinc plays a key role in enhancing the overall performance of the metallo-hydrogels, providing valuable insights into their potential applications in the field of materials science and biomaterials. Through our investigations, it was also observed that Fmoc-protected peptides were found to yield stiffer hydrogels due to favorable π–π stacking effects. In addition to their enhanced mechanical performance, Zn­(II)-containing hydrogels exhibited broad-spectrum antibacterial activity against both *Staphylococcus aureus* and *Escherichia coli*, consistent with the known bacteriostatic role of zinc ions and their frequent use in antimicrobial biomaterials and wound-healing formulations.

## 2 Materials and Methods

### 2.1 Materials for Peptide Synthesis

Fmoc-rink amide AM resin (resin 0.78 mmol g^–1^) was purchased from Merck. The series of Fmoc protected amino acids including leucine, isoleucine, valine, lysine, and histidine, as well as diisopropylethylamine (DIPEA) were purchased from Iris Biotech; (2-(1H-benzotriazol-1-yl)-1,1,3,3-tetramethyluronium hexafluorophosphate) (HBTU) from Acros-Fischer; trifluoroacetic acid (peptide grade, TFA) from Fluorochem Limited; triisopropylsilane (TIS) from TCI Europe; piperidine (99%) from Alfa Aesar; diethyl ether (Et_2_O) and dichloromethane (DCM) from ChemSolute; dimethylformamide (DMF) from Fischer Chemical, and acetic anhydride (Ac_2_O) from Sigma-Aldrich. Kaiser test reagents were prepared according to literature, potassium cyanide and pyridine were acquired from Merck and Acros-Fischer, respectively (reagent A), n-butanol and ninhydrin from Alfa Aesar (reagent B), and phenol and n-butanol from Alfa Aesar and Acros-Fischer, respectively, (reagent C).^42^ For gel preparation, PBS (phosphate-buffered saline 10×) was used as purchased from Alfa Aesar. Collagen Type I was purchased from MP Biomedicals. Milli-Q reagent water (18.2 MΩ cm, 25 °C, Millipore, UK) was used for all experiments.

### 2.2 Peptide Synthesis and Purification

Ac-LIVKHH-NH_2_ (**P1**) and Fmoc-LIVKHH-NH_2_ (**P2**) peptides were synthesized based on Fmoc/Boc solid phase peptide chemistry (Figure S1).^43,44^ In brief, Fmoc-rink amide AM resin (0.78 mmol g^–1^) was weighed out, and the beads were allowed to swell for 1h in DMF. Fmoc deprotection was achieved with a mixture of 20% v/v piperidine in DMF, which was added to the resin and left to agitate for 15 min. After thorough washing with DMF, 2 equiv of HOBt and HBTU were dissolved in DMF and combined. Another 2 equiv of the initial protected amino acid, predissolved in DMF, were introduced to the mixture, followed by the addition of 2 equiv of DIPEA to the same solution, allowing 20 s for activation. Subsequently, this solution was added to the resin and left to agitate for 45 min. The resin was washed with DMF and subjected to a series of deprotection and coupling reactions with the desired amino acids. All the reactions were performed at 25 °C, and the couplings were monitored via Kaiser test. After a thorough washing with DMF, a DMF solution of 12 equiv of Ac_2_O and DIPEA was added (capping) to prevent side reactions of the side groups, and the mixture was incubated for 30 min. To obtain the **P1**, the Fmoc group of the final amino acid was deprotected. Then, the *N*-terminus of **P1** was acetylated using a 5-times excess of Ac_2_O and DIPEA. In case of **P2** the *N*-terminus remained intact with the Fmoc group still attached. The resin was subsequently washed with DMF and DCM and allowed to dry before the peptide cleavage step using a mixture of 94% TFA, 3% Milli-Q water, and 3% TIS. The solvents were reduced under an argon atmosphere, and Et_2_O was added to precipitate the peptide. The peptides were isolated through several centrifugations, washed with Et_2_O, and dried under reduced pressure. The peptides were then lyophilized in Milli-Q water with 0.01% TFA and analyzed by a high-performance liquid chromatography system coupled to a mass spectrometer (HPLC-MS, Thermo scientific, Ultimate 3000 HPLC system coupled with maXis UHR-TOF). The purity of the desired peptides ranged between 95 and 99%, therefore, no further purification was necessary. ESI-MS spectra were measured on a Bruker maXis UHR-TOF equipped with an ESI electrospray ionization chamber in positive mode. Yield: **P1**: 287 mg (57.4%); **P2**: 379 mg (75.8%), ESI-MS: Calculated for C_37_H_62_N_12_O_7_ (786.98, **P1**) and C_50_H_70_N_12_O_8_ (967.19, **P2**) ([M+H^+^]^+^), found *m*/*z* 787.52 (**P1**) and 967.55 (**P2**).

### 2.3 Nuclear Magnetic Resonance (NMR)

^1^H and ^13^C NMR spectra were recorded in D_2_O on a Bruker BioSpin AV NEO 600 MHz spectrometer. During sample preparation, 5 mg of peptide powder was dissolved in D_2_O. Later, 600 μL of the solution was transferred into an NMR tube.

**P1** ^1^H NMR (100% D_2_O): δ = 8.56 (dd, 2H), 7.29 (dd, 2H), 4.65 (t, 2H), 4.28 (dd, 2H), 4.19 (d, 1H), 4.06 (d, 1H), 3.24 (m, 2H), 3.16 (m, 2H), 2.98 (t, 2H), 1.85 (m, 1H), 1.67 (m, 5H), 1.59 (m, 4H), 1.42 (m, 1H), 1.36 (m, 1H), 1.21 (m, 1H), 0.88 (m, 21H) ppm. ^13^C NMR (100% D_2_O): δ = 174.76, 174.13, 173.53, 173.32, 173.00, 171.23, 133.58, 133.47, 128.18, 128.09, 117.29, 117.23, 115.47, 59.32, 57.93, 53.19, 52.63, 52.42, 52.28, 39.67, 39.10, 35.63, 30.35, 29.99, 26.44, 26.39, 26.15, 24.46, 24.26, 22.12, 21.87, 21.45, 21.00, 18.25, 17.82, 14.64, 9.62 ppm.

**P2** ^1^H NMR (100% D_2_O): δ = 8.61 (d, 2H), 7.92 (d, 2H), 7.69 (dd, 2H), 7.50 (t, 2H), 7.43 (q, 2H), 7.29 (d, 2H), 4.64 (q, 3H), 4.54 (m, 1H), 4.32 (t, 1H), 4.25 (m, 1H), 4.10 (d, 1H), 4.00 (, 2H), 3.23 (td, 2H), 3.1 (, 2H), 2.95 (t, 2H), 1.48 (, 1H), 1.33 (m, 2H), 1.43 (m, 4H), 1.67 (m, 4H), 1.81 (m, 1H), 1.97 (m, 1H), 0.86 (d, 4H), 0.81 (s, 13H), 0.72 (d, 1H) ppm. ^13^C NMR (100% D_2_O): δ = 173.70, 173.49, 173.17, 171.35, 163.06, 157.72, 144.04, 143.63, 141.06, 133.69, 133.57, 128.30, 128.16, 127.6, 125.07, 125.00, 124.85, 120.24, 118.89, 117.25, 117.38, 117.32, 115.59, 113.94, 66.75, 59.47, 59.26, 58.25, 57.84, 53.73, 53.30, 52.53, 52.39, 47.36, 47.18, 39.93, 39.20, 35.65, 30.39, 30.21, 30.02, 26.50, 26.24, 24.64, 24.37, 24.20, 22.21, 22.11, 18.34, 17.91 ppm.

### 2.4 Hydrogel Preparation

All hydrogels were prepared using either Milli-Q water (with the pH adjusted with 750 mM NaOH solution to approximately pH = 7) or phosphate-buffered saline (PBS 10×, pH 7.4) at room temperature (25 °C). Briefly, preweighed peptide powder of **P1** and **P2** was dissolved in a total volume of 1 or 2 mL (depending on the measurement) in Milli-Q water, and left for several minutes to allow for hydration and dissolution. Then 0.5 equiv of Zn­(OAc)_2_ was added to the solution. The vial was vortexed and sonicated for 10–20 s to achieve a homogeneous solution. The pH was then adjusted to approximately 7 using 750 mM NaOH solution. Similarly, buffer-containing hydrogels were prepared using 10% buffer in Milli-Q water and then added 0.5 equiv of Zn­(OAc)_2_. The glass vial, where hydrogels were prepared, was vortexed for 10–20 s to achieve an even distribution of the buffer. Similar hydrogels were also prepared in the absence of Zn­(OAc)_2_. The vials were kept on the bench, and hydrogel formation was initially observed over time via the vial inversion method and later was verified through rheology (see section 3.3). Collagen hydrogels were prepared by dissolving collagen type I in Milli-Q water to a concentration of 15 mg mL^–1^ for **P1** and 19 mg mL^–1^ for **P2**. PBS was then added to achieve a 10% PBS concentration in the final solution. The solution was titrated to pH 7.4 using 0.1 M of NaOH. Gelation occurred at 37 °C in 1 h.^27^

### 2.5 Circular Dichroism (CD)

CD measurements were conducted on a Chirascan Plus (Applied Photophysics) spectrometer using a quartz cuvette with an optical path length of 0.01 mm. Peptide solutions with concentrations ranging from 2 to 50 mM were dissolved in Milli-Q water and monitored at room temperature (25 °C) within a wavelength range of 190–280 nm in steps of 1 nm and with a spectral bandwidth of 1 nm. The spectra were acquired as an average of 5 runs for each sample. A baseline correction of all spectra was performed in Milli-Q water, depending on the measurement. CD signals were further normalized to the molar ellipticity value.

### 2.6 Viscoelastic Characterization of Peptide Hydrogels

Rheology measurements were performed using an HR-2 Discovery Hybrid Rheometer (TA Instruments) with 25 mm diameter aluminum plates using parallel geometry and connected with a Peltier plate to control the temperature, with a gap distance of 450 μm. The viscoelastic properties and mechanical stiffness of the peptide hydrogels were monitored via oscillatory frequency sweeps. To determine the linear viscoelastic region (LVR) of each hydrogel, we performed strain sweep measurements at a fixed frequency of 1 Hz while incrementally increasing the applied strain from 0.1% up to 100% (Figures S16 and S17). The LVR was identified as the region in which the storage modulus (*G*′) remained constant. All subsequent frequency sweeps were performed at 0.1% strain, which lies well within the LVR for all samples. The hydrogels were prepared using 15 mg mL^–1^ of **P1** and 19 mg mL^–1^ of **P2** in the presence of 0.5 equiv of Zn­(OAc)_2_. The samples were formed into silicon molds (Figure S13), yielding similar transparent hydrogel discs with a 5 mm diameter. Three replicates of each hydrogel were prepared to ensure measurement accuracy, and results are reported as mean ± SD. Tan δ values (*G*″/*G*′) were calculated using the same plateau region to allow comparison of elastic versus viscous contributions. Viscoelasticity measurements were performed via frequency-sweep. The frequency sweep covered a spectrum of angular frequencies spanning from 0.1 to 100 rad s^–1^ while maintaining a constant strain at 0.1%. Mechanical stiffness was assessed by plotting the elastic modulus, *G*′, against angular frequency, ω. Shear recovery analysis was carried out with sequential strain steps of 0.05% for 180 s, 100% for 180 s, and 0.05% for 600 s, repeated consecutively three times. These incremental steps facilitated the disruption of the hydrogel, inducing liquefaction, and enabled the monitoring of *G*′ and *G*″ recovery dynamics.

### 2.7 Transmission Electron Microscopy

Prior to transmission electron microscopy (TEM) measurements, samples were lyophilized, suspended in DCM, and drop-casted onto 200-mesh copper grids coated with carbon film. TEM was measured at the Electron Microscopy Facility at IST (Austria) using a Phillips Tecnai 12 (120 kV) TEM equipped with a CMOS TVIPS TemCam-F216 camera. The resulting pictures were processed with Gatan Micrograph software and analyzed with TVIPS EM Measure β 0.85.

### 2.8 Fourier Transform Infrared Spectroscopy (FTIR)

Spectra were acquired from 3800 to 400 cm^–1^ (with 128 scans) using a Bruker Tensor-37 FTIR spectrometer equipped with a diamond single-bounce attenuated total reflectance (ATR) sample cell. The hydrogel samples were lyophilized overnight under a high vacuum before the measurement. The powder of the peptides obtained after synthesis was also measured as a reference.

### 2.9 Bacteria Growth Inhibition Zone: Agar Diffusion Assay

A single colony of *S. aureus* (CCUG 19434) was grown in Tryptic Soy Broth (TSB) and *E. coli* (NCTC 13476) in Luria–Bertani (LB) medium overnight at 37 °C. The overnight culture was diluted in 1:50 ratio in the same growth medium and incubated until OD_600_ reaches mid log phase (0.6–1.0). 200 μL of the culture was spread evenly on TSB and LB agar plates and incubated overnight at 37 °C. On the following day, the hydrogels were prepared freshly. Briefly, 18 mg/mL peptide and 9 mg/mL Zn­(OAc)_2_ solutions were prepared in Milli-Q. Peptide solution was incubated at room temperature for 15 min and sonicated for few minutes. In a glass vial, 500 μL peptide solution, 450 μL Zn and 50 μL 10× sterile PBS was mixed. After the addition of 10× PBS, the gelation started immediately. This semiliquid mixture was carefully transferred onto a piece of silicone mold placed on hydrophobic glass slides and left at room temperature for several minutes to allow gelation to occur. Once the gels had formed, they were gently placed onto agar plates with *S. aureus* and *E. coli* and then incubated for 24 h at 37 °C

## 3 Results and Discussion

### 3.1 Synthesis, Peptide Design and Hydrogelation of Zn-Responsive Hydrogels

Amphiphilic peptides have been extensively investigated over the years for their role in self-assembly.^9,45–47^ Therefore, an amphiphilic sequence of amino acids was selected, consisting of one lipophilic (Leu, Ile, Val) and one hydrophilic (Lys, His) end. Two short amphiphilic peptides, Ac-LIVKHH-NH_2_ (**P1**) and Fmoc-LIVKHH-NH_2_ (**P2**) were designed based on their binding affinity to Zn­(II) via their two histidine moieties. These histidine moieties can be deprotonated under physiological conditions, yielding imidazoles capable of coordinating with divalent zinc.^48,49^ Each peptide coordinates to two of the four sites available to the typically tetrahedral coordinated Zn­(II), leading to a metal-peptide cross-linking node.^25,50–52^ (Scheme 1)

**Scheme 1:**
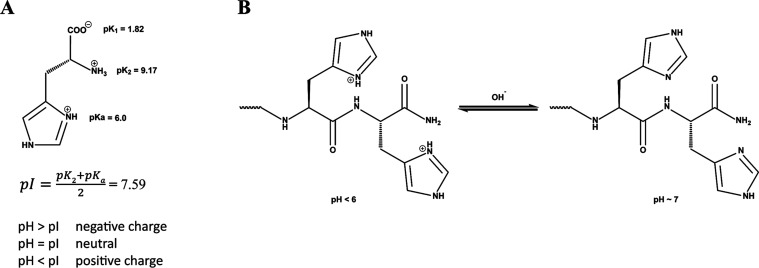
(A) Histidine Protonation, and (B) Protonation or Deprotonation of Histidine Moieties at Different pH Values of Peptide Solution

Beyond enabling metal coordination, the ionization state of histidine itself plays a significant role in governing supramolecular organization. Recent studies have shown that histidine-rich short peptides undergo charge-regulated self-assembly, where deprotonation at physiological pH promotes β-sheet enrichment, fibril maturation, and progressive mechanical stiffening of the hydrogel network.^53,54^ This pH-dependent mechanism aligns with our observations: deprotonation of the histidines in **P1** and **P2** above pH 6 reduces electrostatic repulsion, facilitates His–Zn–His cross-linking, and enables the rapid formation of the fibrillar and sheet-like architectures observed in TEM and FTIR analyses (Sections 3.2 and 3.3).

Furthermore, *N*-terminus modification on one of the peptides is expected to exert a greater influence on hydrogel formation, particularly due to the presence of the Fmoc group in comparison with its acetylated analogue. The Fmoc moiety, known for its π–π stacking effects, enhances hydrophobic interactions and promotes the formation of stiffer hydrogels.^19^

The minimum gelation concentration (MGC) was determined through the dissolution of **P1** and **P2** in Milli-Q water at physiological conditions at different peptide concentrations. For **P1**, this was 15 mg mL^–1^, while **P2** displayed a minimum gelation concentration of 3 mg mL^–1^. This 5-fold lower MGC for **P2** is attributed to the Fmoc group boosting hydrophobic and π-stacking interactions, consistent with prior findings that aromatic capping groups greatly promote peptide self-assembly. To test if the peptides indeed show stimuli responsiveness to Zn­(II) ions, Zn­(OAc)_2_ was added to a peptide solution and the pH was adjusted to pH ∼ 7. Peptides **P1** and **P2** with Zn­(II) ions present are denoted as **P1**_**Zn**_ and **P2**_**Zn**_ respectively. For **P1**, which typically requires 1 h to form a gel in Milli-Q water, the addition of Zn­(II) significantly accelerates the process, reducing the gelation time to approximately 20 min. Similarly, while a solution of 3 mg mL^–1^ of **P2** in Milli-Q water takes about 20 min to form a hydrogel, the observed gelation time can be reduced to 5 min in the presence of 0.5 equiv. Zn­(OAc)_2_, clearly demonstrating the influence of Zn­(II) ions on the gelation behavior of **P2**.

Stiffer hydrogels, formed via higher peptide concentrations, required a shorter gelation period. For example, 19 mg mL^–1^ of **P2**_**Zn**_ took only a few seconds to form a hydrogel, compared to 20 min for 3 mg mL^–1^ (Figure 1). Further, Zn­(II)- containing hydrogels are also stiffer than those without Zn­(II). Finally, changes in the mechanical properties are also observed based on the used solvent, where PBS-buffered solutions typically result in stiffer gels when compared to gels prepared just in Milli-Q water. The difference in their mechanical properties was quantified and further assessed via oscillatory rheology (Section 3.3).

**Figure 1:**
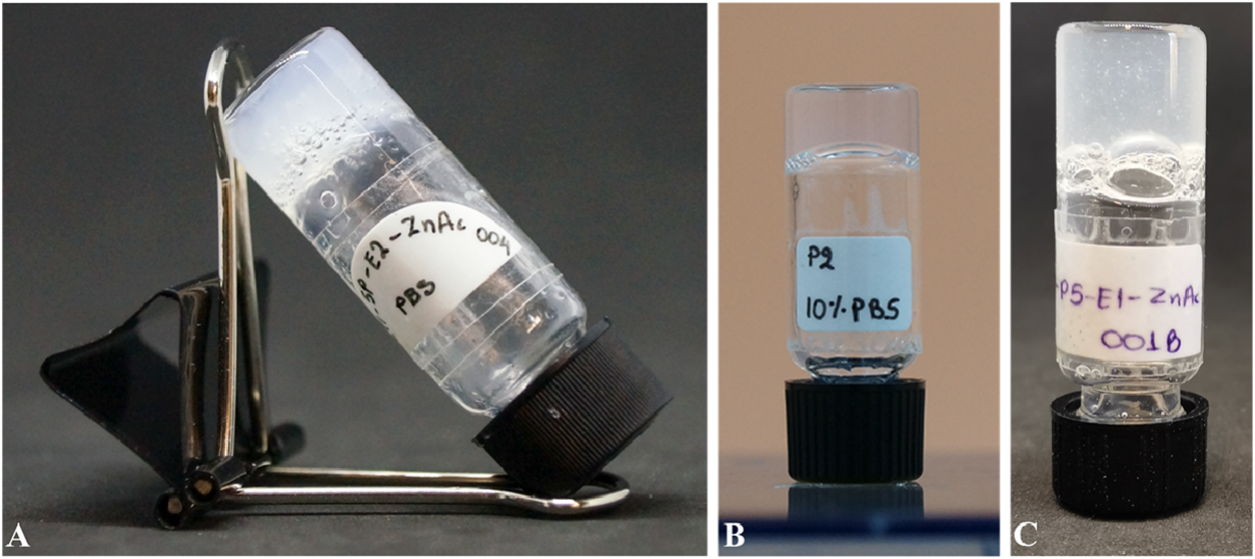
Water based hydrogels of (A) **P2** at 19 mg mL^–1^ with 1 equiv of Zn­(II) in 10% PBS; (B) **P2** at 19 mg mL^–1^ in 10% PBS; (C) **P2** at 19 mg mL^–1^ with 0.5 equiv of Zn­(II) in aqueous solution. Note: Small bubbles visible in figure C occurred after the rapid gelation of **P2**_**Zn**_ upon addition of Zn­(II). Air pockets introduced during brief vortexing become trapped as the hydrogel solidifies within seconds.

### 3.2 Structural Conformation of Stimuli-Responsive Hydrogels and Microstructure

**P1** and **P2** each contain two histidines (Scheme 1B), which are positively charged below pH 6 (His­(imidazole) p*K*_a_ = 6.0). Under these conditions, the resulting electrostatic repulsion of peptides prevents self-assembly. Conversely, above pH 6, the deprotonation of the histidine moieties removes the electrostatic repulsion and facilitates Zn­(II) binding, leading to rapid self-assembly and hydrogel formation (Scheme 2).^55^

**Scheme 2:**
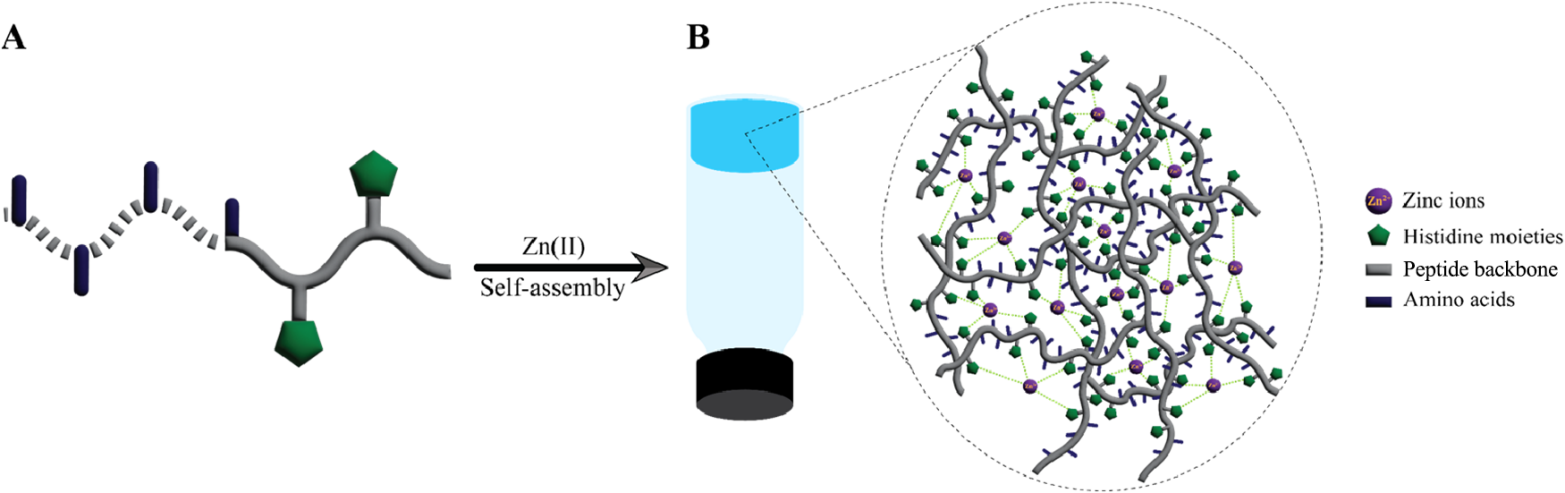
(A) Part of Peptide Monomer, and (B) Schematic of Hydrogel Formation in the Presence of Zn­(II) that Acts as Coordinative Crosslinking Agent Favoring His-Zn-His Bond Formation

The secondary conformation of the peptides in the samples with and without Zn­(II) was analyzed by circular dichroism. In the absence of Zn­(II), both **P1** (Figure 2A) and **P2** (Figure 2B) display a random coil conformation (negative peak between 190 and 200 nm) at low concentrations (0.5 mM, 4.86 mg mL^–1^). As the concentration is increased, these conformations change, with **P1** transitioning to an α-helix conformation at 50 mM, evidenced by the replacement of the negative peak at 197 nm by two at 207 and 225 nm, whereas at 30 mM **P2** gives a strong positive peak at 199 nm, with no negative peaks at longer wavelength, indicating a conformation related to that of a β-sheet. In the presence of 0.5 equiv of Zn­(OAc)_2_ (Figure 3C,D), increasing the peptide concentration had little effect on the conformations of either of the peptides, with both giving CD signals consistent with random coil, as well as displaying some other small peaks which are not straightforward to assign. This suggests that the coordination cross-links restrict the conformational space of the peptide and take precedence over prior conformational tendencies. In general, the presence of Zn­(II) may lead to Zn-imidazole coordination, which stabilizes the random coil structure, and may hinder the formation of a well-defined secondary structure. Thus, both peptides appear to have a relatively disordered, but flexible coil structure. This does not imply a lack of supramolecular order; rather, the peptides still self-assemble (the solutions form gels), but their polypeptide backbones remain relatively disordered due to the multivalent binding of Zn­(II). Similar observations have been reported in other metal–peptide systems where metal ligation interrupts the formation of canonical secondary structures in favor of amorphous cross-linked networks.^14,56^

**Figure 2:**
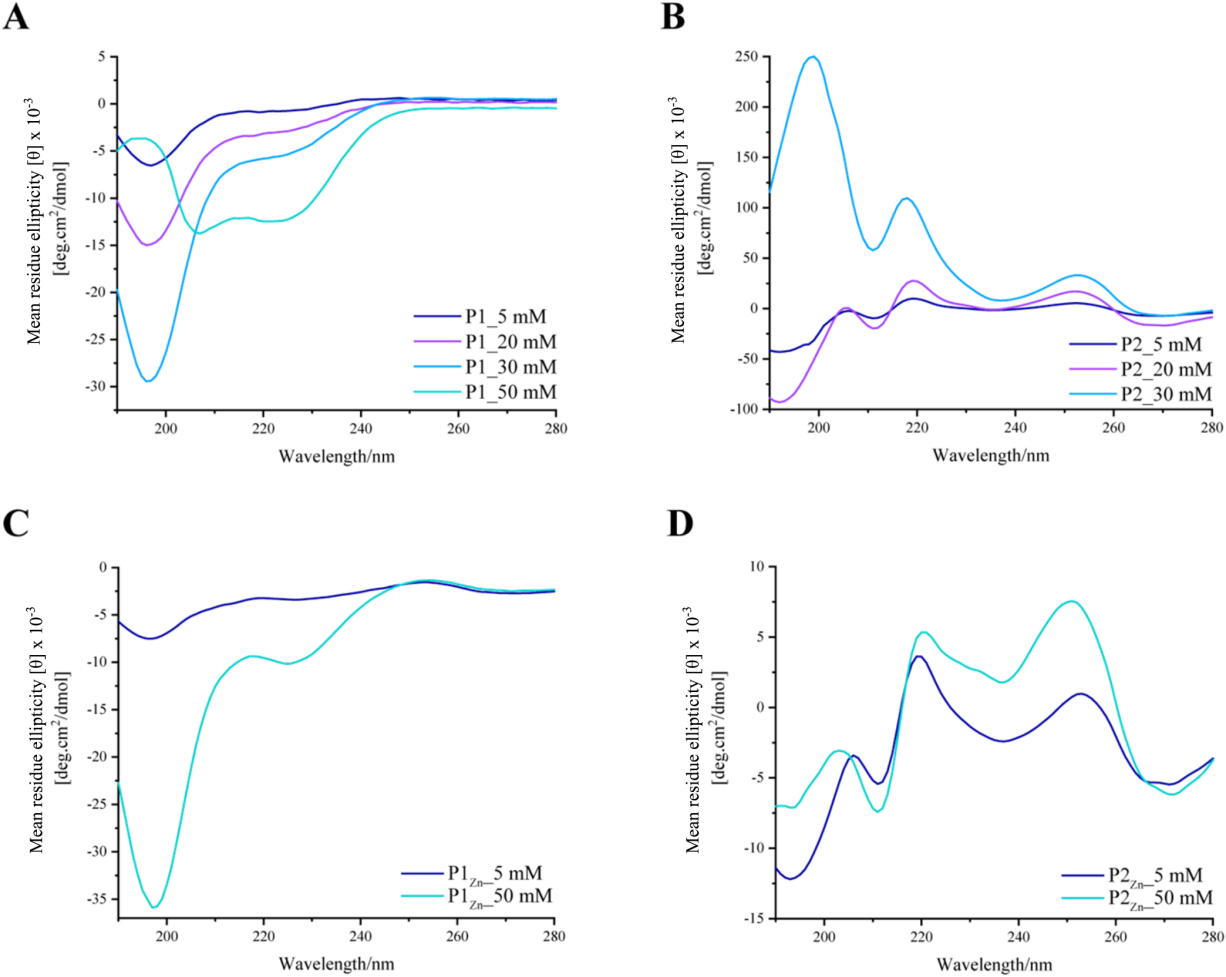
Circular dichroism (CD) scans of increasing concentration of **P1** (left column) and **P2** (right column) were conducted to monitor the changes in the secondary structure of peptides under various conditions. In graph A (**P1**) & graph B (**P2**), the scans were performed in aqueous solution at pH 7, showing a change in their structural conformation from random coil to α-helix and to β-sheet, respectively. In graph C (**P1**) and graph D (**P2**), the scans were conducted in an aqueous solution at pH 7 with 0.5 equiv of Zn­(OAc)_2_.

**Figure 3:**
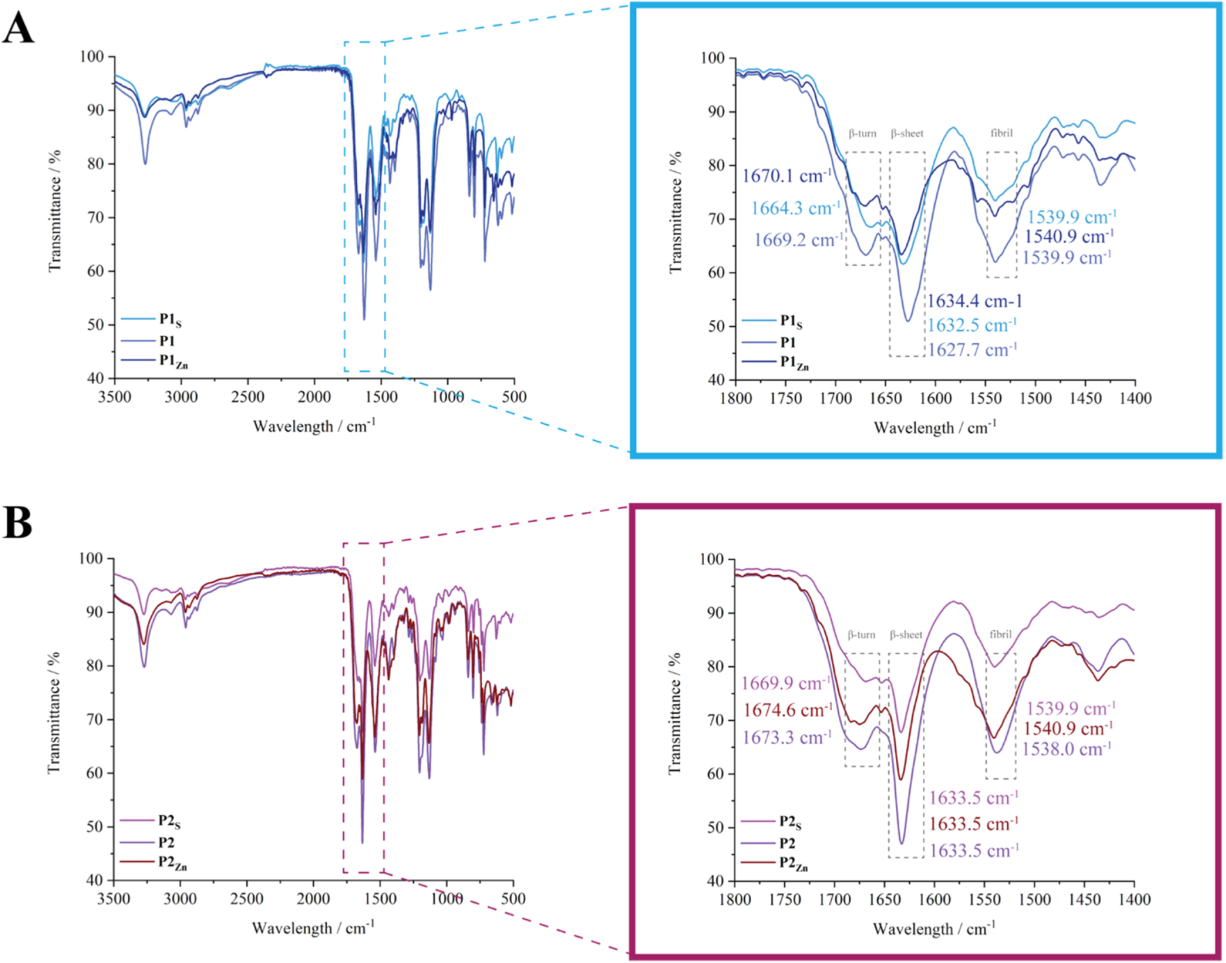
FTIR spectra of lyophilized peptide gels with Zn­(II). (A) P1 series: (dark blue) **P1**_**Zn**_ at 15 mg mL^–1^ with 0.5 equiv of Zn­(II), (blue) **P1** at 15 mg mL^–1^, and (light blue) **P1**_**S**_ peptide powder as obtained after synthesis. (B) P2 series: (purple) **P2**_**Zn**_ at 19 mg mL^–1^ with 0.5 equiv of Zn­(II), (light purple) **P2** at 19 mg mL^–1^, and (pink) **P2**_**S**_ peptide powder as obtained after synthesis. The zoomed-in regions (right panels) show the characteristic amide I bands corresponding to β-turn, β-sheet, and fibril structures.

The secondary structure of the peptides was further investigated using FT-IR spectroscopy. Lyophilized samples of **P1**, **P1**_**Zn**_, **P2**, and **P2**_**Zn**_ were analyzed and compared with their respective peptides as obtained as powders immediately after synthesis (**P1**_**S**_ and **P2**_**S**_). In the Amide I region, **P1**, **P1**_**Zn**_, and **P1**_**S**_, exhibit a set of characteristic peaks, including a weak and broad peak at approximately 1664.3–1670.1 cm^–1^ and a strong, sharp peak at around 1627.7–1634.4 cm^–1^, both indicative of β-sheet structures (Figure 3A). Additionally, a strong and relatively broad peak is observed in the Amide II region at around 1539.9–1540.9 cm^–1^, which is characteristic of fibril structures (Figure 3A).^29,39,57^

Specifically, β-sheets are characterized by a sharp, intense Amide I band around 1620–1640 cm^–1^ for antiparallel β-sheets and 1670–1695 cm^–1^ for parallel β-sheets, with the Amide II region appearing between 1520 and 1580 cm^–1^.^16,58,59^ The FT-IR data qualitatively demonstrates that **P1**_**S**_, which did not have an opportunity to undergo self-assembly, has a less intense peak in the Amide I region compared to the self-assembled **P1** and **P1**_**Zn**_, corresponding to β-sheet and β-turn structures. Overall, the intensity of peaks in the Amide I region of **P1** and **P1**_**Zn**_ compared to **P1**_**S**_ is slightly decreased at the β-turn region and is stable at the β-sheet region, while, in the Amide II region the intensity increased, indicating self-assembly into fibrils (Figure S10).

Similarly, **P2, P2**_**Zn**_, and **P2**_**S**_ samples display peaks in the Amide I, with a weak and broad peak at about 1669.9–1673.3 cm^–1^ and a strong, sharp peak at 1633.5 cm^–1^, also indicative of β-sheet structures (Figure 3B). Another strong and relatively broad peak is observed in the Amide II region at approximately 1538.0–1540.9 cm^–1^, characteristic of fibril structures (Figure 3B). A similar trend in peak intensity is observed for **P2** (Figure S11).

These findings are consistent with the TEM micrographs discussed below, which reveal stacked fibrillar and in some cases, sheet-like structures in **P1**, **P1**_**Zn**_, **P2**, and **P2**_**Zn**_ (Figure 4). The FT-IR spectra therefore suggest that the peptide hydrogels **P1**, **P1**_**Zn**_, **P2**, and **P2**_**Zn**_, as well as the peptides themselves, **P1**_**S**_ and **P2**_**S**_, predominantly adopt fibrillar structures, along with β-sheet conformations (Figures 3A, 4, S10 and S11). The presence of Zn­(II) does not disrupt β-sheet formation but appears to enhance intermolecular packing, as reflected by slightly sharper Amide I and II bands, suggesting stronger hydrogen bonding and cross-strand interactions. Together with the CD data, these results highlight the complementary nature of the techniques: while CD provides insights into solution-state conformations, FTIR of lyophilized gels captures the intermolecular β-type organization characteristic of the solid state. Thus, the apparent random-coil features observed in CD spectra and the β-sheet signatures in FTIR are not contradictory but rather reflect distinct structural states within the hierarchical assembly process.

**Figure 4:**
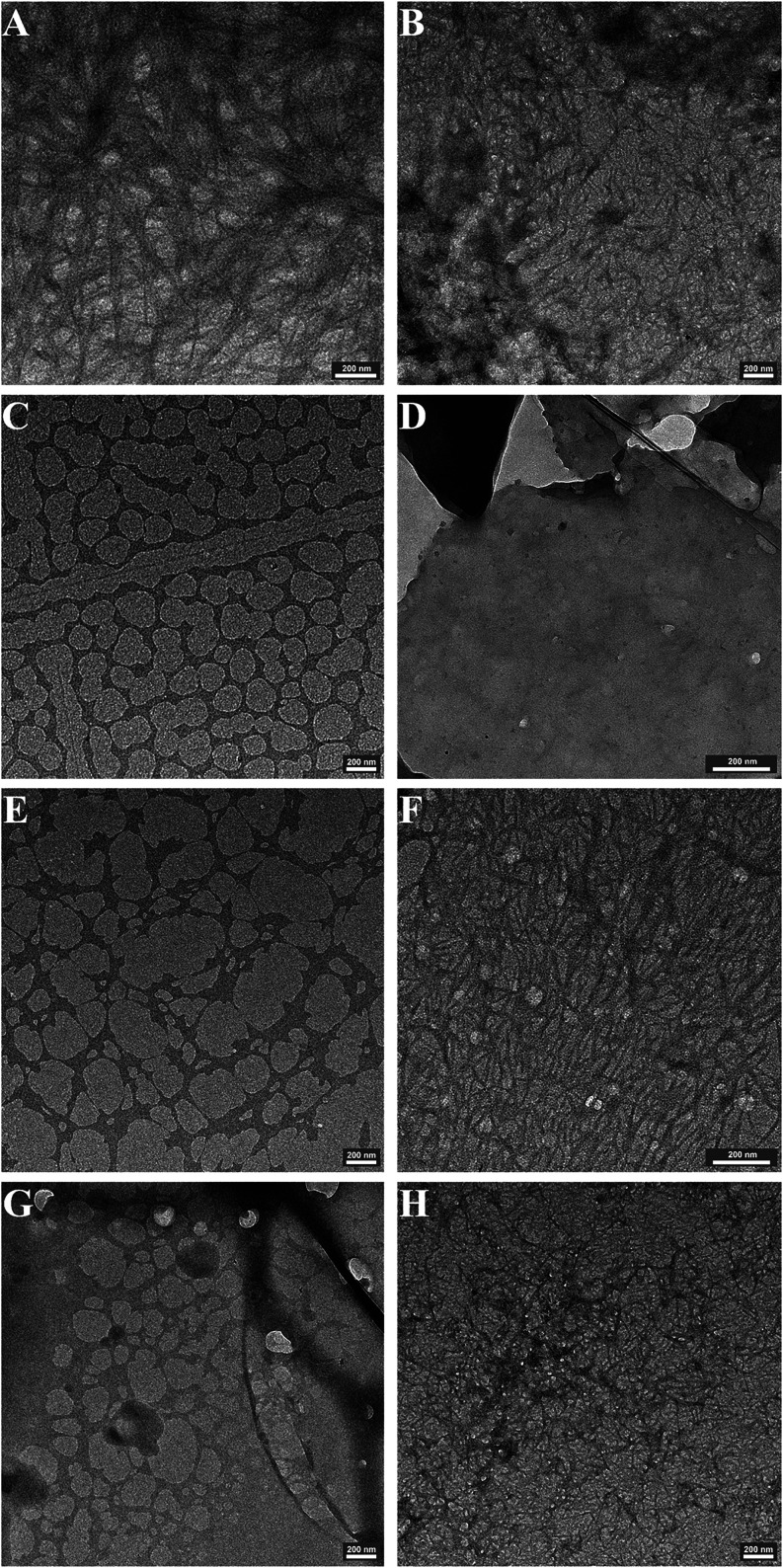
TEM micrographs in 200 nm scale (A) **P1** at 4 mg mL^–1^; (B) **P2** at 5 mg mL^–1^; (C) **P1**_**Zn**_ at 4 mg mL^–1^ with 0.5 equiv of Zn­(II); (D) **P2**_**Zn**_ at 5 mg mL^–1^ with 0.5 equiv of Zn­(II); (E) **P1** at 15 mg mL^–1^; (F) **P2** at 19 mg mL^–1^; (G) **P1**_**Zn**_ 15 mg mL^–1^ with 0.5 equiv of Zn­(II), and (H) **P2**_**Zn**_ at 19 mg mL^–1^ with 0.5 equiv of Zn­(II).

The differences between the CD and FTIR results arise because these techniques probe distinct hierarchical levels of structure and therefore provide complementary information. CD reflects the solution-state conformation of individual polypeptide backbones, where Zn­(II) coordination limits conformational flexibility and leads to predominantly random-coil–like conformation for **P1**_**Zn**_ and **P2**_**Zn**_. FTIR, measured on lyophilized hydrogels, instead shows the intermolecular hydrogen-bonding and supramolecular packing within the assembled networks, revealing β-sheet- and fibril-associated Amide I and II bands. Thus, peptides may retain conformational disorder in solution while simultaneously assembling into β-sheet-rich fibrillar networks upon aggregation. This behavior is consistent with prior metal–peptide systems in which metal coordination disrupts canonical backbone folding while enhancing intermolecular order.^14^ Similar divergences between solution-state disorder and solid-state β-sheet organization have been documented in several metal–peptide systems, where metal coordination reduces backbone flexibility and suppresses canonical secondary structures observable by CD, yet simultaneously promotes intermolecular β-sheet stacking detected by FTIR or X-ray methods. This decoupling of intramolecular and supramolecular order has been reported for Zn­(II)- and Cu­(II)-coordinated short peptides, histidine-containing amphiphiles, and metal-triggered dipeptide gelators.^60–62^

To further elucidate the supramolecular architecture of the peptide hydrogels and confirm the morphology suggested by FTIR, transmission electron microscopy (TEM) was assessed to visualize the nanoscale organization of the peptide hydrogels with and without Zn­(II). The TEM analysis provided direct evidence of how zinc coordination influences the morphology, revealing transitions from extended fibrillar networks to more compact or sheet-like structures, thus bridging the molecular-level spectroscopic observations with the macroscopic gel properties.

For this, two concentrations were chosen, one close to the minimum gelation concentration of **P1**, **P1**_**Zn**_, **P2**, and **P2**_**Zn**_ and a higher concentration, at which both peptides form stable hydrogels. The same concentration was used for the rheology experiments. First, the peptide was dissolved in Milli-Q water, the pH was adjusted with NaOH to physiological conditions (pH = 7.4) and the peptide mixture was allowed to undergo self-assembly for 1 h in the case of **P1** and **P1**_**Zn**_, while **P2** and **P2**_**Zn**_ were only incubated for 5 min. Hereby the differences in gelation time are based on our observation that P1 takes a significantly longer time to form solid gels. To allow TEM measurements and to maintain the self-assembled structures, peptide hydrogels were shock-frozen in liquid N_2_ followed by lyophilization overnight. To obtain low peptide concentrations on the TEM grid, a small amount of lyophilized sample was resuspended in DCM and drop-casted onto the TEM grid.

At low peptide concentrations, **P1** (Figure 4A) and **P2** (Figure 4B) form an extended fibrous network of stacked fibers. Upon the addition of Zn­(II), **P1**_**Zn**_’s (Figure 4C) fibril network is disrupted forming a flat sheet-like structure. **P2**_**Zn**_ (Figure 4D) on the other hand forms an even more pronounced sheet-like structure. At higher peptide concentrations, **P1** forms a distorted sheet-like structure (Figure 4E) which does not seem to change significantly when Zn­(II) is present (Figure 4G). However, the Zn­(II) affects the morphology and the connectivity within the sample. **P2** forms a fibrous network of finer fibers (Figure 4F), while **P2**_**Zn**_ forms a network of stacked, and more numerous nanofibers (Figure 4H).

Overall, the presence of Zn­(II) at physiological pH facilitates Zn-imidazole coordination and peptide self-assembly, stabilizing the secondary structure in **P2**_**Zn**_ samples. This coordination may disrupt the formation of flexible, continuous well-defined fibrous secondary structures. However, it promotes the formation of more robust, shorter, yet well-defined fibrous networks. Zn­(II) acts as a cross-linker, potentially resulting in a more rigid and stable network, which can be observed as more consistent or thicker fibers in the TEM images.

### 3.3 Assessment of Viscoelastic Properties of Self-Assembled Peptide Hydrogels

#### 3.3.1 Rheology Experiments

Mechanical stiffness and viscoelastic character of the peptide hydrogels were evaluated through oscillatory rheology experiments. Hydrogels were prepared at a consistent peptide concentration of 15 mg mL^–1^ for **P1** and 19 mg mL^–1^ for **P2** and pH ∼ 7–7.4. A series of experiments was conducted to quantify the differences in hydrogel behavior between aqueous and 10% phosphate-buffered saline (PBS) solutions with (**P1**_**Zn**_, **P2**_**Zn**_) and without Zn­(II) (**P1**, **P2**). The peptide concentration was carefully selected to ensure hydrogel formation in both cases.

Storage (*G*′) and loss (*G*″) moduli for all samples were quantified through an oscillatory rheology frequency sweep. Both peptides exhibit enhanced viscoelastic properties in 10% PBS in the presence of Zn­(II) when compared to the samples without Zn­(II). As mentioned above, we postulate that Zn­(II) can act as a cross-linker agent under physiological pH, facilitated by cross-strand interactions (His-Zn-His) with the peptide, leading to improved mechanical properties of hydrogels. In the absence of Zn­(II), *G*′ values are significantly lower (Table 1), consistent with the slower and more loose formation of hydrogels.

**Table 1:** Values of Storage (*G*′) and Loss (*G*″) Modulus of Frequency Sweep Plots of P1 and P1Zn at 15 mg mL^–1^, and P2, and P2Zn at 19 mg mL^–1^

		P1/P1_Zn_			P2/P2_Zn_		
	samples’ characteristics	storage M. (*G*′)/MPa	loss M. (*G*″)/MPa	tan (delta)	storage M. (*G*′)/MPa	loss M. (*G*″)/MPa	tan (delta)
25 °C	Milli-Q, pH 7	no gelation	no gelation		0.150 (±0.00152)	0.019 (±0.00088)	0.121 (±0.01275)
	10% PBS	no gelation	no gelation		0.004 (±0.00029)	0.0005 (±0.00006)	0.113 (±0.01670)
	Zn(II), Milli-Q	0.012 (±0.00043)	0.001 (±0.00002)	0.080 (±0.00093)	0.155 (±0.09410)	0.008 (±0.00813)	0.053 (±0.00316)
	Zn(II), 10% PBS	no gelation	no gelation		0.246 (±0.02366)	0.022 (±0.00148)	0.085 (±0.00369)
37 °C	Milli-Q, pH 7	no gelation	no gelation		0.152 (±0.00924)	0.018 (±0.00036)	0.126 (±0.01668)
	10% PBS	no gelation	no gelation		0.011 (±0.00113)	0.0011 (±0.00015)	0.095 (±0.00204)
	Zn(II), Milli-Q	0.393 (±0.01447)	0.035 (±0.00182)	0.091 (±0.00599)	0.174 (±0.08014)	0.008 (±0.00287)	0.048 (±0.01057)
	Zn(II), 10% PBS	no gelation	no gelation		0.304 (±0.00684)	0.026 (±0.00034)	0.089 (±0.00423)

In general, **P1** shows a lower tendency toward gelation compared to **P2**, due to the lack of the Fmoc moiety. At peptide concentration of 15 mg mL^–1^ for **P1** and 19 mg mL^–1^ for **P2** and pH ∼ 7–7.4, only **P2** (but not **P1**) hydrogels could be formed for both MQ and PBS solutions. With 0.5 equiv of Zn­(II), **P1**_**Zn**_ hydrogels formed for Milli-Q water but not PBS solutions (Figure 5A, Table 1). The presence of Zn­(II) aids hydrogel formation via coordinative binding by the histidine moieties, but PBS may lower the effective Zn­(II) concentration via phosphate binding, preventing hydrogel formation.^32,63,64^

**Figure 5:**
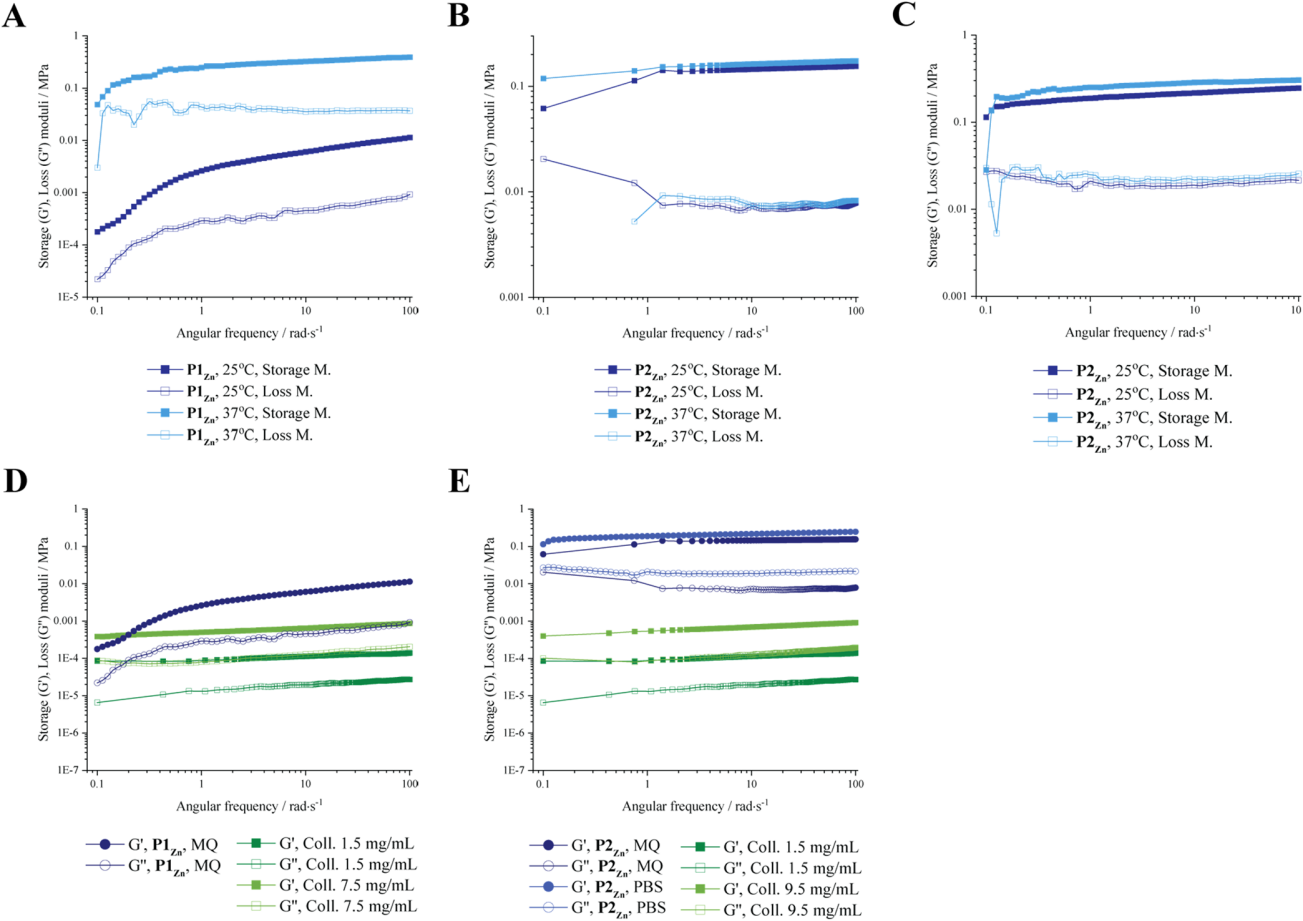
Frequency sweep rheological measurements of peptide-based hydrogels in the presence and absence of Zn­(II). (A) **P1**_**Zn**_ and (B) **P2**_**Zn**_ hydrogels prepared in aqueous solution were evaluated at room temperature (25 °C) and physiological conditions (37 °C) to assess the effect of Zn­(II) on their viscoelastic properties. (C) Frequency sweeps of **P2**_**Zn**_ hydrogels in 10% PBS at 25 and 37 °C demonstrate the influence of Zn­(II) in saline conditions, while **P1** did not form stable gels within 24h under these conditions and was therefore excluded from the measurements. (D, E) Comparative frequency sweeps of **P1**_**Zn**_ (D) and **P2**_**Zn**_ (E) hydrogels with their respective collagen analogues show differences in stiffness and frequency dependence.

**P2** hydrogels show stiffer viscoelastic properties in Milli-Q water rather than PBS solutions in the absence of Zn­(II) (Figure S14 A and B, respectively). However, with 0.5 equiv of Zn­(II), the **P2**_**Zn**_ hydrogels in Milli-Q water have similar mechanical strength to those in the absence of Zn­(II), but demonstrate a slight increase in mechanical strength only when increasing the temperature from 25 to 37 °C (Figure 5B, Table 1). **P2** hydrogels in 10% PBS, though, become significantly stiffer and show no loss of mechanical strength or stiffness when increasing the temperature from 25 to 37 °C, but rather a slight increase in *G*′ to 0.304 MPa values (Figure 5C, Table 1).

The effect of Zn­(II) strongly depends on both peptide type and medium/buffer. For **P2** in Milli-Q water, the Fmoc group promotes β-sheet-rich fibrillization and a dense network, as such introducing 0.5 equiv. Zn­(II) only modestly affects *G*′ (0.150 → 0.155 MPa). On the other hand, in 10% PBS the nonmetalated **P2** gel is much weaker (*G*′ ≈ 0.004 MPa), indicating that ionic strength and specific ion effects disrupt optimal fibril–fibril connectivity. Under these buffered conditions, Zn­(II) acts as an additional, orthogonal cross-linker via His–Zn–His bridges, converting the weak **P2** network into a highly reinforced metallo-hydrogel (*G*′ up to ∼0.3 MPa). The acetylated analogue **P1**, which lacks the Fmoc π-stacking motif, shows a different behavior: at 15 mg mL^–1^ it does not form a mechanically stable gel in either Milli-Q or 10% PBS within 24 h, and only the Zn­(II)-containing **P1**_**Zn**_ sample in Milli-Q reaches measurable *G*′ values, which remain well below those of **P2**_**Zn**_ under comparable conditions. In 10% PBS, even **P1**_**Zn**_ fails to gel, consistent with the combination of weaker intrinsic self-assembly and phosphate-mediated depletion of free Zn­(II). These trends highlight that Fmoc-driven hydrophobic/π–π interactions in **P2** provide a robust primary scaffold that can be further reinforced by metal coordination, whereas **P1** relies predominantly on Zn­(II) cross-links and is therefore far more susceptible to medium composition.

Furthermore, across all conditions, *G*′ values consistently exceeded *G*″ values, indicating the presence of viscoelastic properties characteristic of a gel. To further quantify the relative elastic and viscous contributions, tan δ (*G*″/*G*′) values were extracted from the plateau region of each frequency-sweep curve. Across all gels that formed (Table 1), tan δ remained low (≈ 0.05–0.12), consistent with predominantly elastic, cross-linked networks. Such low tan δ values are characteristic of stable supramolecular hydrogels, confirming that Zn­(II)-mediated coordination not only increases *G*′ but also maintains strong elastic dominance under both aqueous and buffered conditions.^65^ The temperature-induced changes in *G*′ were likewise accompanied by minimal variation in tan δ, indicating that the gels retain their elastic character across the tested conditions. When compared to other reported self-assembling peptide hydrogels (2–6 amino acids), our metal cross-linked hydrogels (**P1**_**Zn**_ and **P2**_**Zn**_) display increased viscoelastic properties by at least 1 order of magnitude, thus clearly demonstrating the potential of peptide hydrogels utilizing metals as cross-linker.^14,21,24,25,27,38,39,41,66–76^ Additionally, the reported *G*′ values and thixotropy (section 3.3.2) are on the verge of being suitable for injection hydrogels, as they exceed the reported limit for injectable biomaterials.^16,70,77,78^

To put the observed values into the context of other biopolymer-based hydrogels, collagen was chosen as a reference compound.^79–81^ Both peptides outperform their collagen analogues, as illustrated in frequency sweep graphs and the above table (Figure 5D,E, Tables 1 and 2), while buffer-based hydrogels exhibit enhanced viscoelastic properties compared with their aqueous analogues. The comparison between **P1**_**Zn**_ (Figure 5D) and **P2**_**Zn**_ (Figure 5E) with collagen demonstrates sufficient elasticity to endure the applied stress and strain as in bioengineering applications.^27,82,83^

**Table 2:** Values of Storage (*G*′) and Loss (*G*″) Modulus* of Frequency Sweep Plots of Varying Concentrations of Collagen

		collagen		
	samples’ characteristics	storage M. (*G*′)/MPa	loss M. (*G*″)/MPa	tan (delta)
25 °C				
	collagen 0.8 wt % (7.5 mg mL^–1^)	0.00086 (±0.00050)	0.00021 (±0.00031)	0.236 (±0.01403)
	collagen 1 wt % (9.5 mg mL^–1^)	0.00090 (±0.00042)	0.00020 (±0.00005)	0.212 (±0.01309)
	collagen 0.2 wt % (1.5 mg mL^–1^)	0.00014 (±0.00029)	0.000027 (±0.00013)	0.204 (±0.02230)

#### 3.3.2 Thixotropy Experiments

The thixotropic behavior of **P2** hydrogels was systematically evaluated through step-strain rheology experiments. The extent of thixotropy is intricately linked to both the duration of applied shear stress and the magnitude of the shear rate.^84^ Both metallo-hydrogels and nonmetallo-hydrogels exhibit remarkable thixotropic properties based on observations of their rapid recovery to the gel-like state. Upon application of a high strain (100%), both samples underwent a gel–sol transition, demonstrated by a sharp decrease in *G*′. When the strain was returned to 0.05%, the hydrogels rapidly reformed, with *G*′ recovering by more than 3 orders of magnitude within seconds (Figure 6). Interestingly, it can also be seen that the presence of Zn­(II) enhanced by an order of magnitude both the *G*′ and *G*″ moduli of the **P2** hydrogel (Figure 6A), without compromising the thixotropic behavior. In addition to the qualitative trends observed in the step-strain profiles, quantitative analysis of the recovery cycles were determined by comparing the restored storage modulus after the final low-strain step to the initial *G*′ value prior to shear. Hydrogels prepared from **P2** in 10% PBS exhibited 91.4% recovery, whereas the Zn­(II)-containing analogue (**P2**_**Zn**_) recovered to 43.2% of its initial modulus under identical conditions (Table S1). These results indicate that Zn­(II) coordination enhances the stiffness of the hydrogel network but partially constrains restructuring after shear, while both materials retain rapid and reproducible thixotropic responses. The remarkable thixotropic behavior and recovery capacity of our peptide hydrogels emphasize their promise as injectable carriers or candidates for 3D printing applications.^77,85^

**Figure 6:**
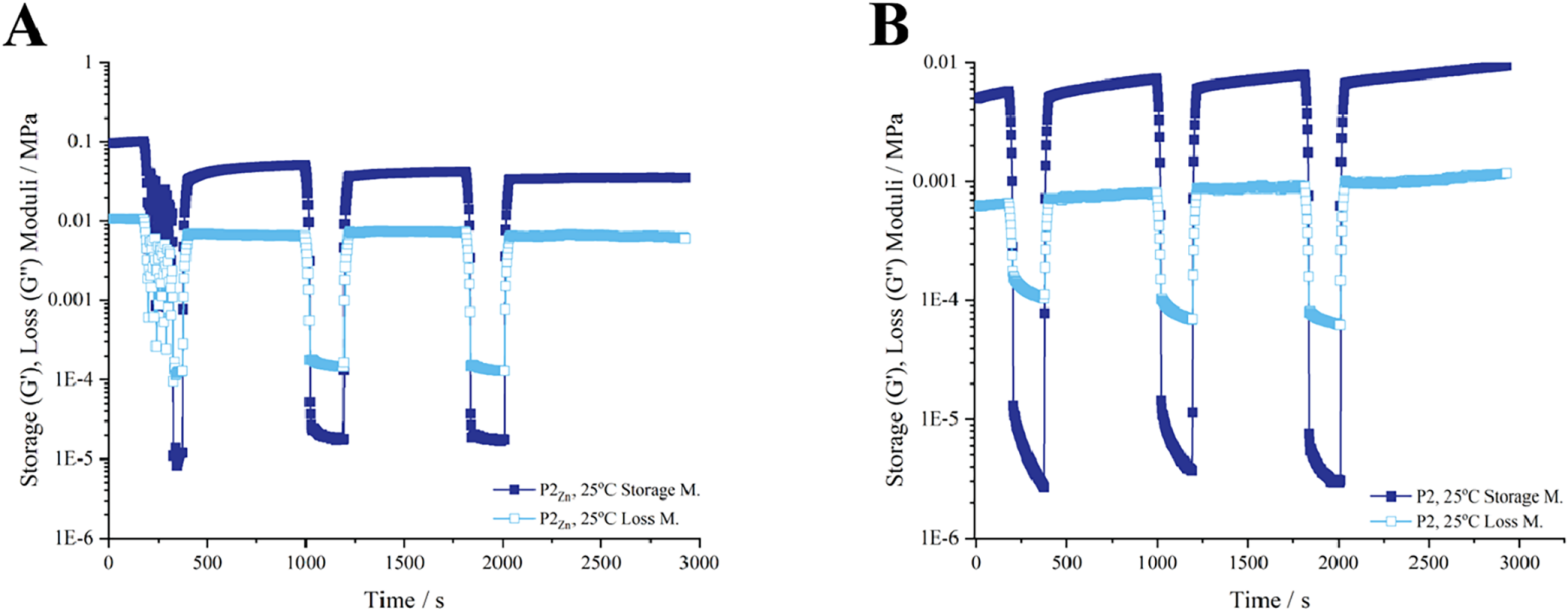
Periodical oscillatory step–strain experiments of P2 hydrogels in 10% PBS at 25 °C, with **P2**_**Zn**_ (A) and without Zn­(II) **P2** (B).

### 3.4 Assessment of Bacteria Growth Inhibition Zone

As an initial, qualitative screen to evaluate whether diffusible Zn­(II) species released from the hydrogels produce observable inhibition zones under standardized conditions. At this diffusion-based screen of antibacterial performance, we evaluated Zn­(II)-loaded peptide hydrogels by agar zone-of-inhibition against *S. aureus* and *E. coli*. This method is commonly used in hydrogel and supramolecular materials research as an early stage assessment, rather than as a quantitative measure of antimicrobial potency. Accordingly, we do not draw conclusions regarding therapeutic relevance or mechanism. This method is widely used and easy to interpret when an active component can elute from the material; zone diameters are typically measured to the nearest millimeter under standardized conditions.^86^ However, for soft, poorly diffusive or contact-active materials, agar diffusion is inherently conservative and may underestimate activity relative to direct contact or time-kill assays—hence we interpret it qualitatively here and use it primarily to confirm release of diffusible antibacterial species. Further, the gelation times can vary, and the resulting gels could be delicate and difficult to handle during transfer to the plates. This affected the consistency and shape of the resulting hydrogels. Under ideal circumstances a growth inhibition zone of Zn­(II)-containing gels is clearly visible around the gels for both *S. aureus* and *E. coli* (Figure S18). The inhibition zone seems to be slightly larger for *S. aureus* than *E. coli*, but overall, the results indicate that the gels display broad-spectrum antibacterial activity. This is consistent with literature that zinc-based systems can inhibit both Gram-positive and Gram-negative bacteria while sometimes showing larger effects against Gram-positive species.^87^ Given the geometric variability that accompanies manual transfer of soft gels, we refrain from comparing absolute halo sizes and instead report representative images to document the effect. In conclusion, although further optimization is needed to improve gel handling and ensure reproducibility, Zn­(II)-loaded peptide hydrogels represent a promising platform for topical antibacterial applications. Their ability to inhibit both Gram-positive and Gram-negative bacteria highlights their potential for use in wound dressings and antimicrobial coatings.

## 4 Conclusions

In this study, we successfully developed two novel amphiphilic peptides, namely Ac-LIVKHH-NH_2_ (**P1**) and Fmoc-LIVKHH-NH_2_ (**P2**), with a specific binding affinity for biologically benign metal salts, particularly zinc. The histidine moieties of the peptides interacted with Zn­(II) in aqueous conditions and physiological pH, forming a coordination polymer, incorporating zinc as a cross-linker, and ultimately leading to metallo-hydrogel formation. Our investigation into the impact of *N*-terminus modifications revealed that Fmoc-protected peptides yield stiffer hydrogels due to favorable π–π stacking effects. Structural analyses, including transmission electron microscopy and FTIR spectroscopy, provided valuable information about the supramolecular architecture, secondary structure, and micro/nanostructure of the hydrogels. Their micro- and nanostructure revealed a flexible fibrous network influenced by observable changes in the presence of Zn­(II). Rheology experiments demonstrated enhanced mechanical stiffness and viscoelasticity, particularly in aqueous and PBS conditions, with **P2**_**Zn**_ outperforming **P1**_**Zn**_. Additionally, both **P1**_**Zn**_ and **P2**_**Zn**_ exhibited promising thixotropic behavior, allowing for rapid recovery after shear-induced liquefaction. Therefore, preliminary agar diffusion assays confirmed that Zn-containing hydrogels release diffusible species capable of generating visible inhibition zones, supporting their potential for further investigation in future biological studies. They also showed that Zn-loaded hydrogels inhibited both *S. aureus* and *E. coli*, indicating a broad-spectrum antimicrobial effect.

These findings highlight the significant role of Zn­(II) as a cross-linker, enhancing the mechanical properties of the hydrogels. The mechanical strength reported for our peptides outperformed other known (metallo)-hydrogels based on short peptides.^14,23–25,37–39,41,72,74,88–91^ This work provides valuable insights into the tunable properties of peptide-based hydrogels through metal coordination, paving the way for their future use in biomedical applications such as wound healing, tissue engineering, and injectable biomaterials. The thixotropic behavior of these peptides also makes them ideal candidates for 3D printing and advanced material science applications.

## Supplementary Material



## Notes

Additional experimental details; NMR spectra (^1^H, ^13^C, COSY, HSQC, HMBC); FTIR spectra; SEM micrographs; rheology data (frequency sweeps, amplitude sweeps, thixotropy); agar diffusion assay images; equation of recovery percentage and table of recovery percentages; additional figures (Figures S1–S18) (PDF)
