# Supplementary material for: Zn(II)-Responsive Peptide Hydrogels with Tunable Mechanical Properties
Source: ACS Omega. 2026 Feb 13;11(7):11971–83. doi: 10.1021/acsomega.5c11025 (PMC12947148; doi:10.1021/acsomega.5c11025)
Supplement: Supplementary file 1 [file ao5c11025_si_001.pdf]

Zn(II)-Responsive Peptide Hydrogels with Tunable Mechanical Properties

Alexia Tialiou[1,2], Christopher J. Serpell[3], Çağrı Özsan[4], Lingcong Ge[1,2], Angelo Frei[4], Jia Min Chin*[5], Bernhard K. Keppler[1,6], Michael R. Reithofer*[1]

[1] Institute of Inorganic Chemistry, Faculty of Chemistry, University of Vienna, Währinger Str. 42, 1090 Vienna, Austria. Email: michael.reithofer@univie.ac.at

[2] Vienna Doctoral School in Chemistry (DoSChem), University of Vienna, Währinger Str. 42, 1090 Vienna, Austria.

[3] School of Pharmacy, University College London, 29/39 Brunswick square, London, WC1N1AX, United Kingdom.

[4] Department of Chemistry, University of York, Heslington, York, YO10 5DD, United Kingdom.

[5] Institute of Functional Materials and Catalysis, Faculty of Chemistry, University of Vienna, Währinger Str. 42, 1090 Vienna, Austria. Email: jiamin.chin@univie.ac.at

[6] University of Vienna and Medical University of Vienna, Research Cluster "Translational Cancer Therapy Research", Währinger Str. 42, 1090 Vienna, Austria.

Contents

1. Chemical structure and characterization 2
1.1 NMR spectra 2
1.2 Fourier transform infrared spectroscopy (FTIR) 6
1.3 Scanning Electron Microscopy 7
2. Rheology measurements 9
2.1 Frequency sweep 9
2.2 Amplitude sweep 10
2.3 Thixotropy 11
3. Bacteria growth inhibition zone assay 11

1. Chemical structure and characterization

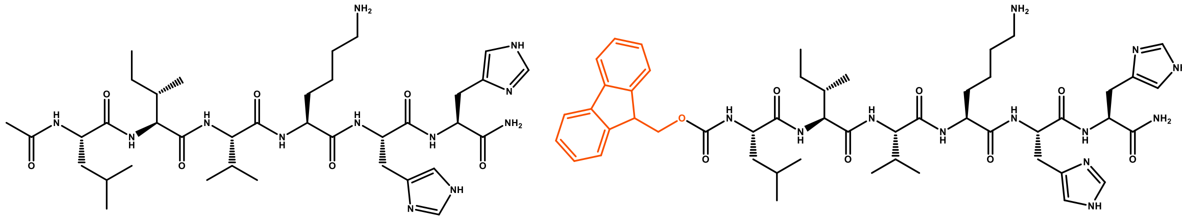

P1 P2

Fig. S1 Chemical structure of Ac-LIVKHH-NH$_2$ (P1) and Fmoc-LIVKHH-NH$_2$ (P2).

1.1 NMR spectra

NMR spectra of Fmoc- and Ac-LIVKHH-NH$_2$ (6.6 mg mL^{-1}) measured in D_2O are below.

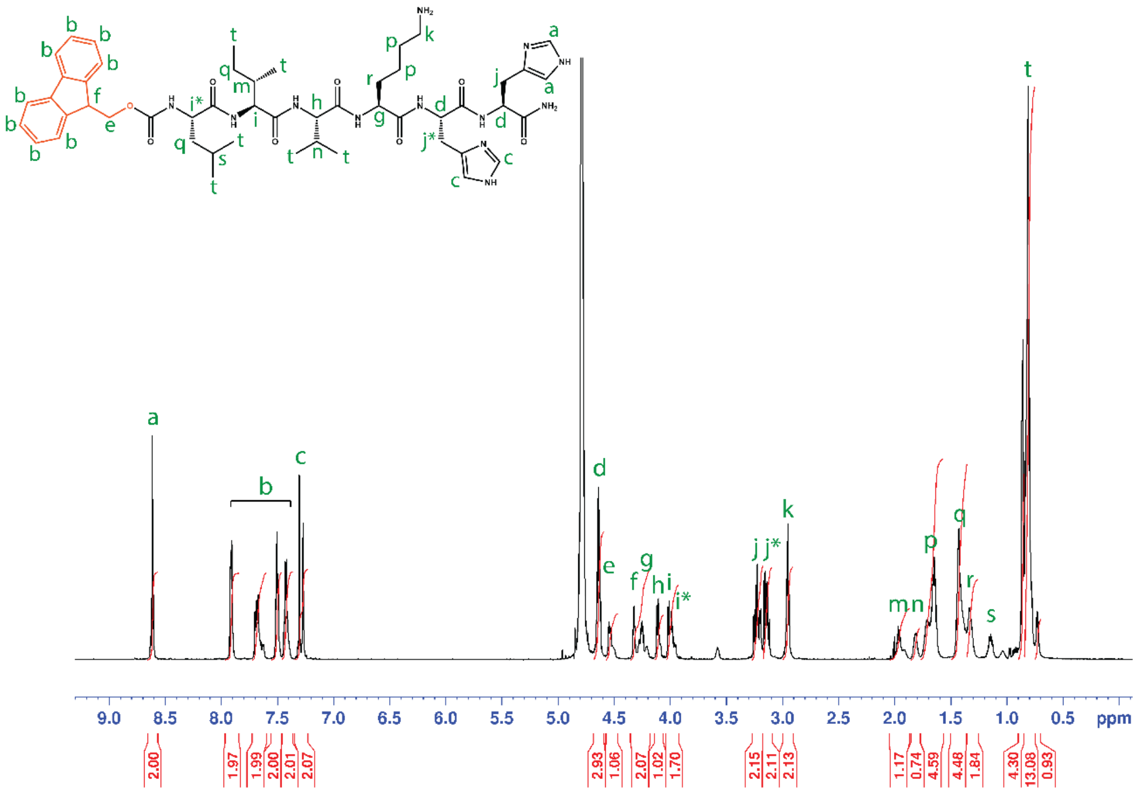

Fig. S2 ^{1}H NMR spectrum of Fmoc-LIVKHH-NH_2.

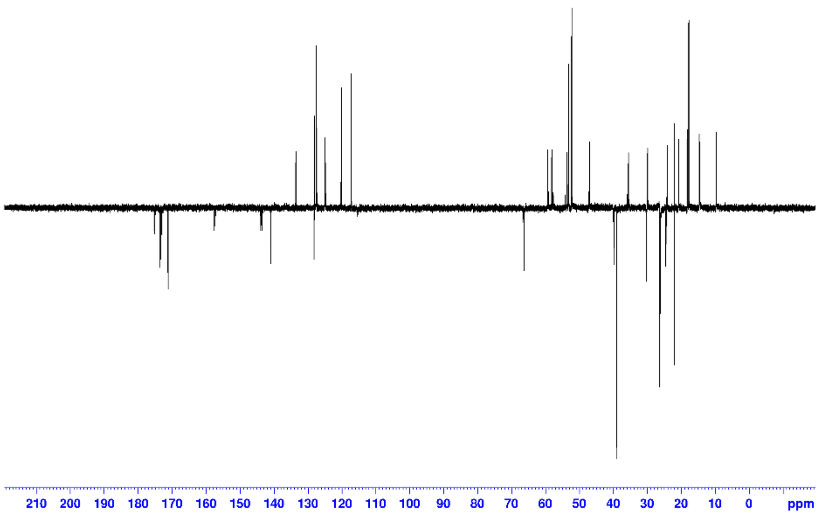

Fig. S3 ^{13}C-DEPTQ NMR spectrum of Fmoc-LIVKHH-NH_2.

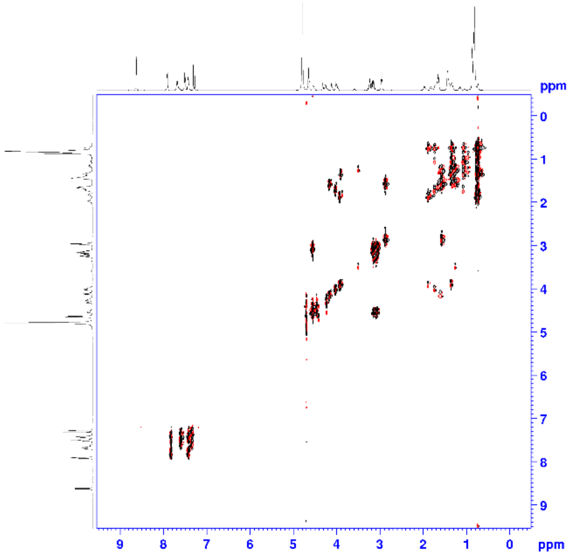

Fig. S4 ^{1}H-^{1}H COSY NMR spectrum of Fmoc-LIVKHH-NH_2.

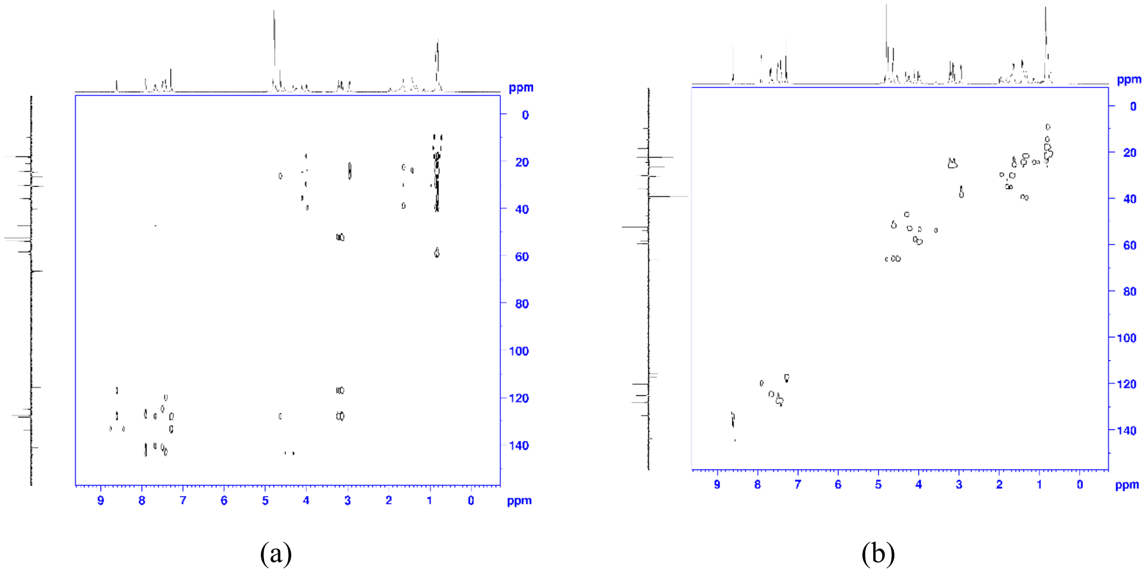

Fig. S5 (a) ^{1}H,^{13}C-HMBC and (b) ^{1}H,^{13}C-HSQC NMR spectra of Fmoc-LIVKHH-NH_2.

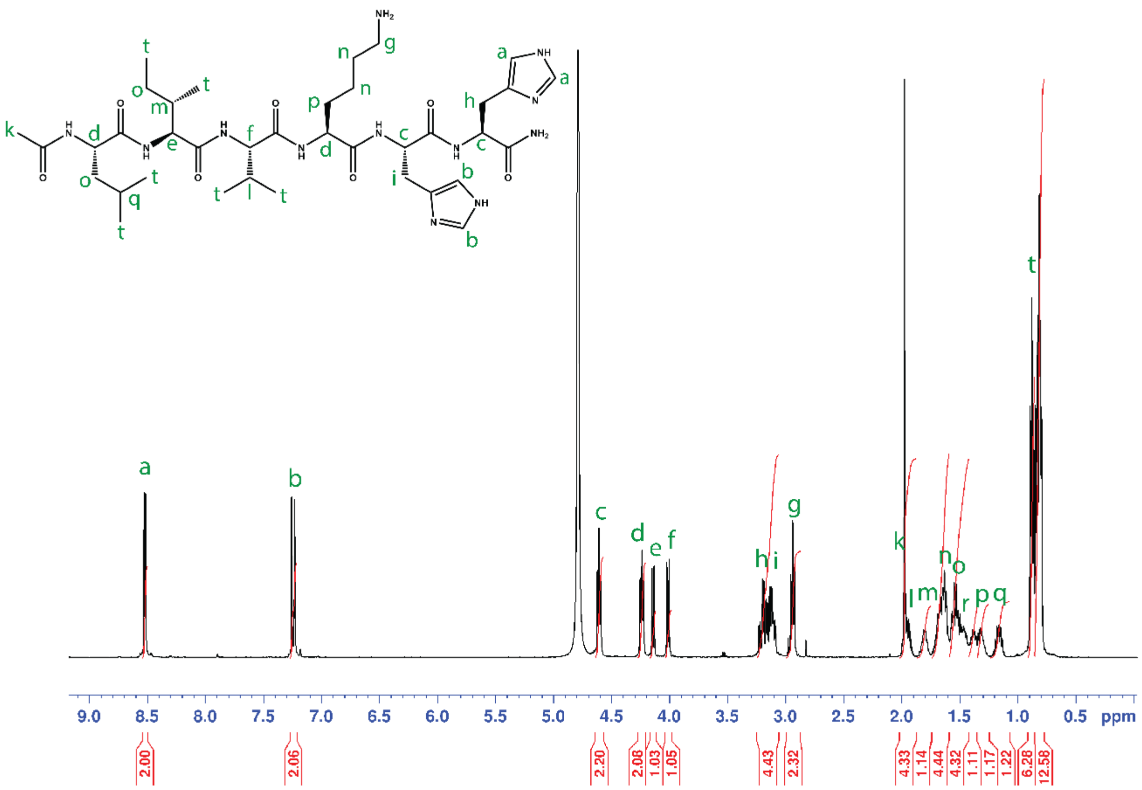

Fig. S6 ^{1}H NMR spectrum of Ac-LIVKHH-NH_2.

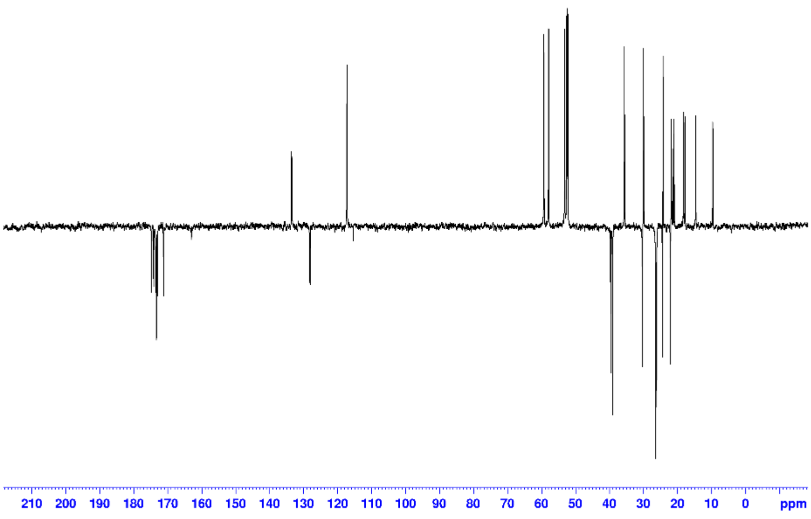

Fig. S7 ^{13}C-DEPTQ NMR spectrum of Ac-LIVKHH-NH_2.

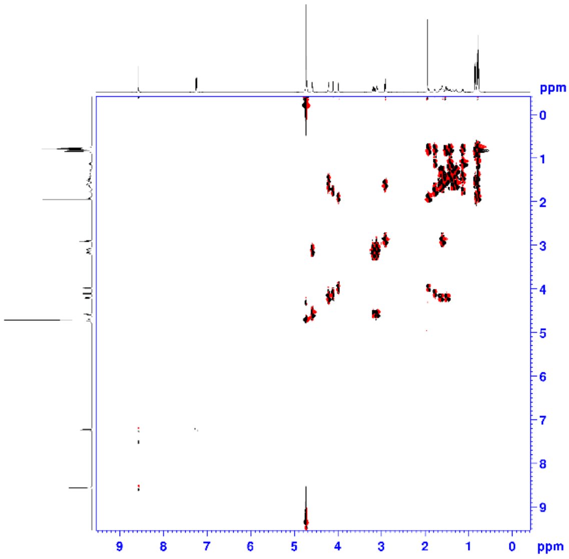

Fig. S8 ^{1}H-^{1}H COSY NMR spectrum of Ac-LIVKHH-NH_2.

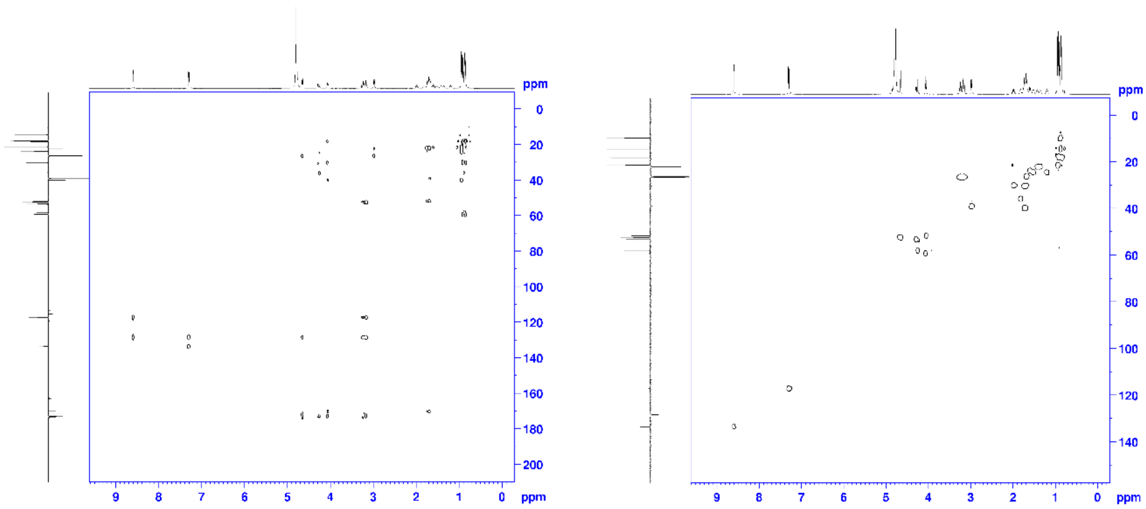

(a) (b)

Fig. S9 (a) ^{1}H,^{13}C-HMBC and (b)) ^{1}H,^{13}C-HSQC NMR spectra of Ac-LIVKHH-NH_2.

1.2 Fourier transform infrared spectroscopy (FTIR)

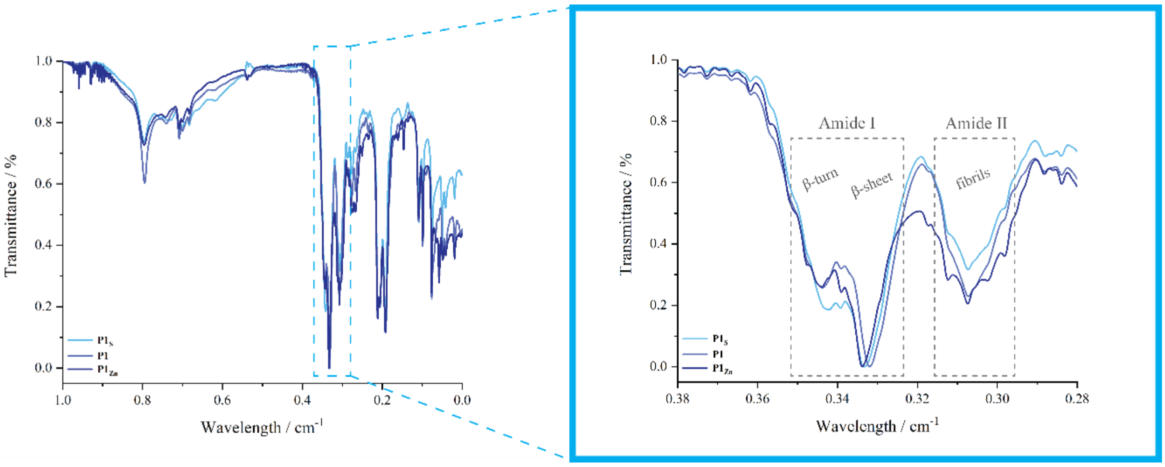

Fig. S10 Normalized FTIR spectrum of lyophilized peptide gels in the presence and absence of Zn(II) of (dark blue) **P1$_{Zn}$** 15 mg mL^{-1} in the presence of 0.5 equiv. of Zn(II), (blue) **P1** 15 mg mL^{-1}, and (light blue) **P1$_S$** peptide powder as obtained after synthesis. The zoomed-in part on the right shows the characteristic regions of β-turn, β-sheet, and fibril regions

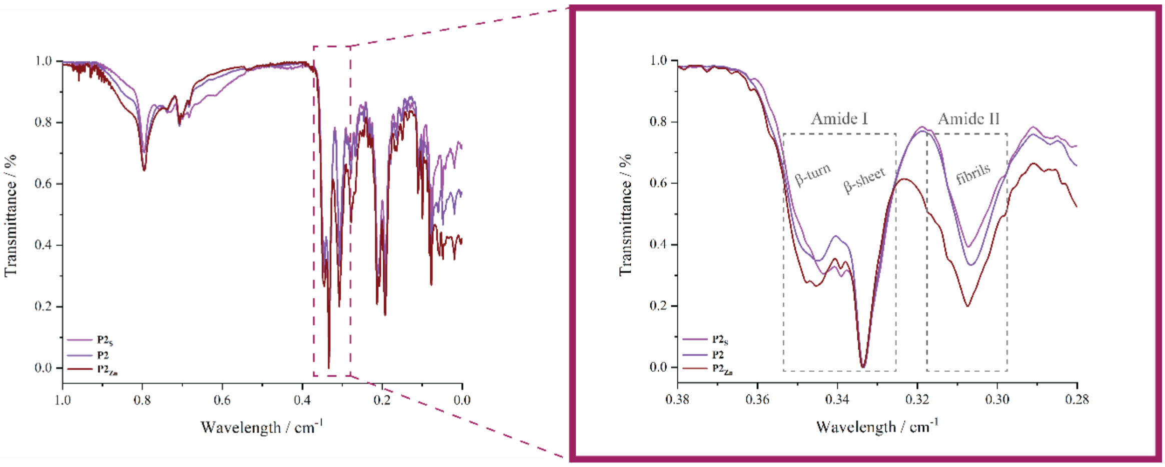

Fig. S11 Normalized FTIR spectrum of lyophilized peptide gels in the presence and absence of Zn(II) of (purple) **P2$_{Zn}$** 19 mg mL^{-1} in the presence of 0.5 equiv. of Zn(II), (light purple) **P2** 19 mg mL^{-1}, and (pink) **P2$_S$** peptide powder as obtained after synthesis. The zoomed-in part on the right shows the characteristic regions of β-turn, β-sheet, and fibril regions.

1.3 Scanning Electron Microscopy

Prior to the SEM measurements, all samples were shock-frozen in liquid nitrogen and lyophilized overnight using a Beta 2-8 LCSplus – Martin Christ lyophilizer. The morphology of the freeze-dried samples was visualized via a Zeiss Supra 55 VP microscope (Faculty Center for Nano Structure Research) with an accelerating voltage between 3 and 5 kV. The samples were carefully fixed onto carbon tape at the top of the aluminum specimen containers and placed on the specimen holder. Subsequently, they were sputter-coated with gold to a thickness of ~ 3 nm (Leica EM SCD050) and positioned at the specimen stage of the SEM. Measurements were performed at a magnification range of 40x to 2000x, using a Secondary Electrons detector.

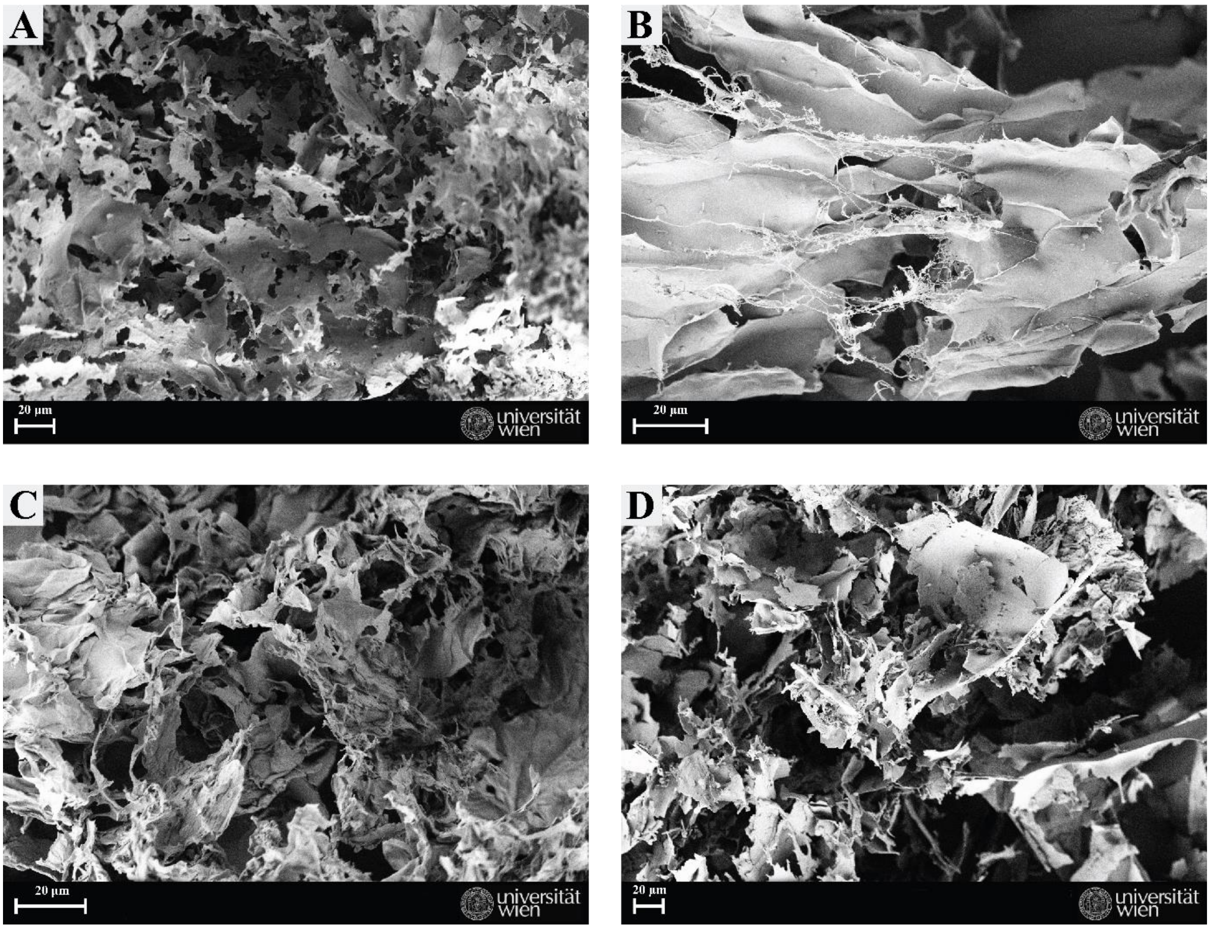

Fig. S12 SEM micrographs depicting the freeze-dried peptide hydrogels containing (A) 19 mg mL^{-1} of **P2** in the presence of 0.5 equiv. of Zn(II); (B) 5 mg mL^{-1} of **P2** in Milli-Q water; (C) 19 mg mL^{-1} of **P2** in 10% PBS, and (D) 4 mg mL^{-1} of **P1** in Milli-Q water.

2. Rheology measurements

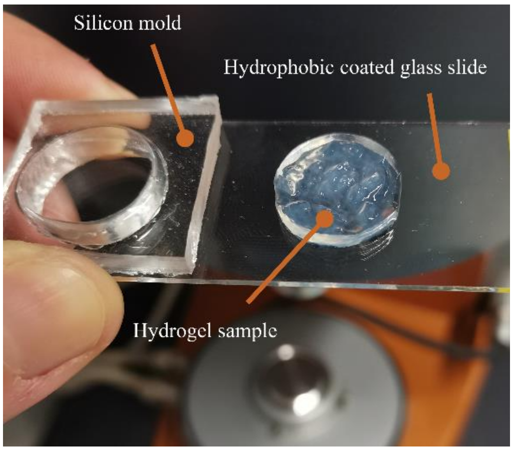

Fig S13 Sample preparation for rheology measurements.

2.1 Frequency sweep

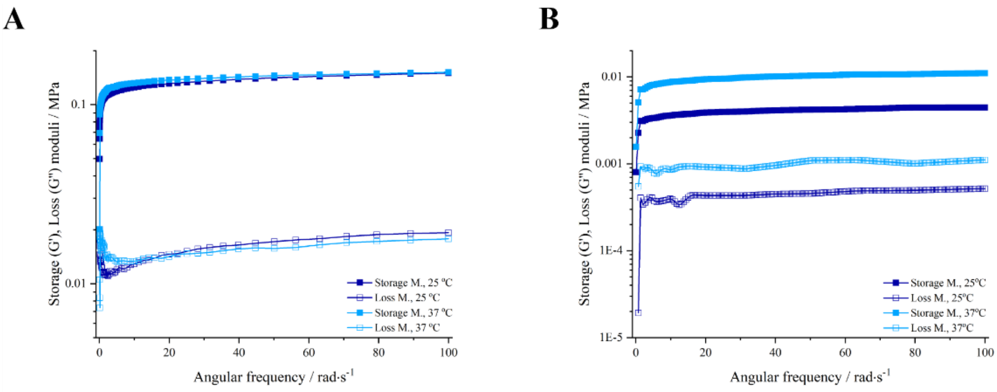

Fig S14 Frequency sweep measurements were conducted for **P2** to study the impact of temperature on the mechanical properties of hydrogels prepared in aqueous (A) and 10% PBS buffer (B) solution at both room temperature (25 °C) and physiological conditions (37 °C) [Ac-LIVKHH-NH_2 did not form hydrogels within 24h in MQ at 19 mg mL^{-1}].

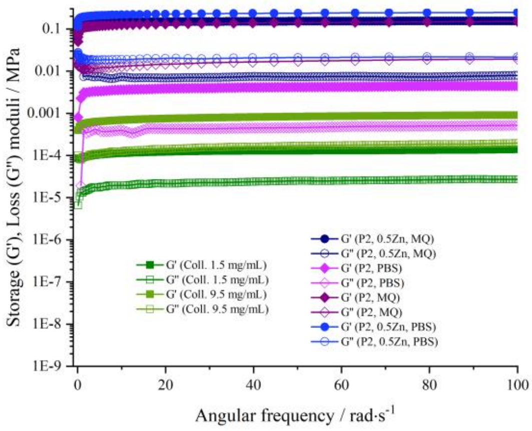

Fig. S15 A cumulative chart of P1, P2 and collagen frequency sweep.

2.2 Amplitude sweep

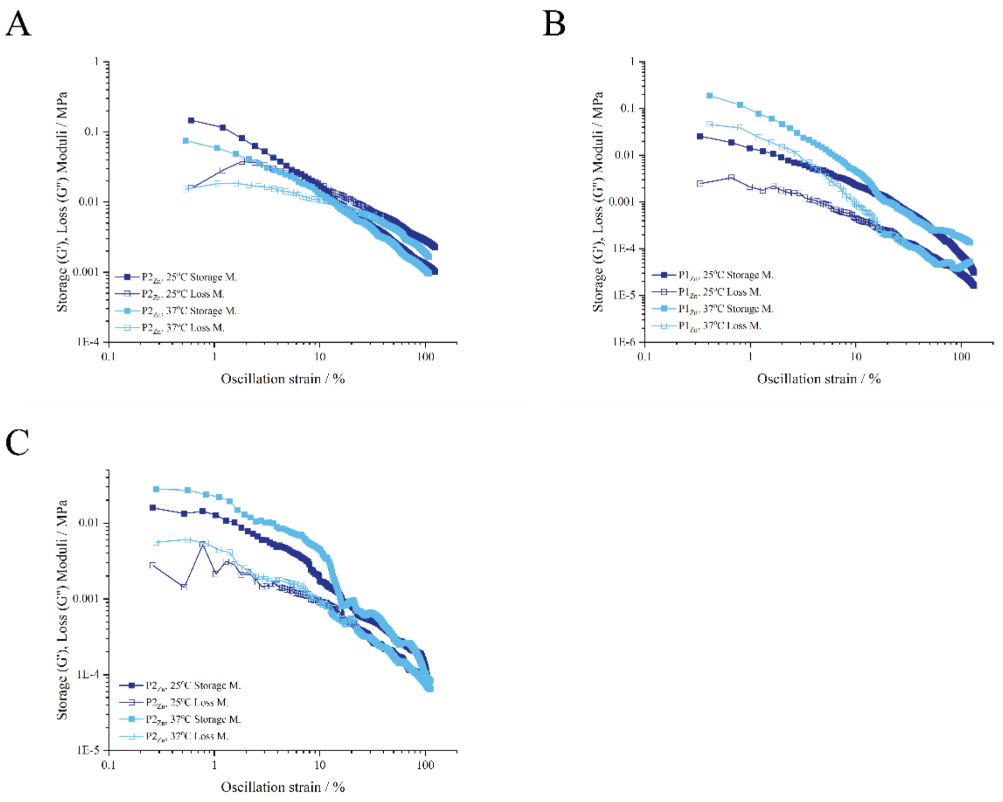

Fig S16 Amplitude (strain) sweep measurements were performed to assess the strain-dependent mechanical stability and to determine the linear viscoelastic region (LVR) of Zn(II)-responsive hydrogels. (A) Amplitude sweeps of $P2_{Zn}$ hydrogels in Milli-Q water at 25 °C and 37 °C, showing the strain-dependent decrease of storage (G′) and loss (G″) moduli. (B) Amplitude sweeps of $P1_{Zn}$ hydrogels in Milli-Q water at 25 °C and 37 °C, demonstrating their lower mechanical stability relative to $P2_{Zn}$. (C) Amplitude sweeps of $P2_{Zn}$ hydrogels prepared in 10% PBS, highlighting the influence of ionic strength under physiological (37 °C) and ambient (25 °C) conditions. In all cases, G′ exceeds G″ within the LVR, confirming gel-like behavior, before both moduli decay at higher strain values.

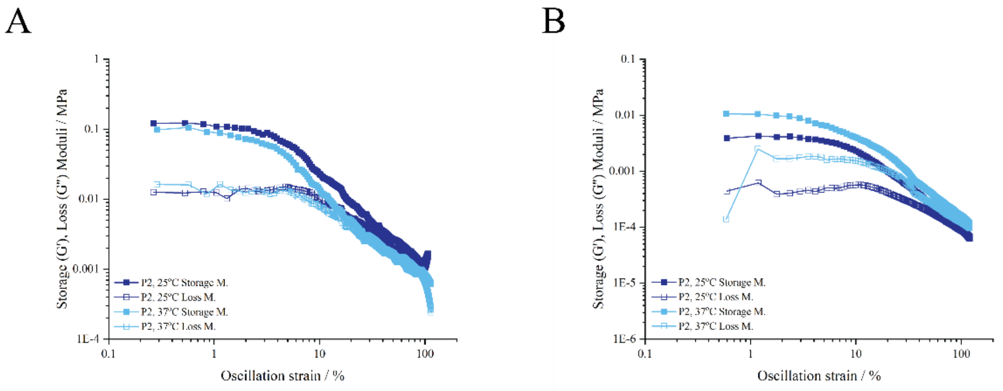

Fig S17 Amplitude sweep measurements of P2 hydrogels were conducted to evaluate their strain-dependent viscoelastic behavior and to identify the linear viscoelastic region. (A) Amplitude sweeps of $P2_{Zn}$ hydrogels in Milli-Q water at 25 °C and 37 °C show a broad LVR followed by a characteristic strain-induced breakdown of the gel network. (B) Amplitude sweeps of $P2_{Zn}$ hydrogels in 10% PBS at both temperatures highlight the enhanced stiffness and resistance to deformation under saline conditions. In all samples, the storage modulus (G′) dominates over the loss modulus (G″) at low strain, confirming elastic, gel-like behavior prior to yielding. [Ac-LIVKHH-NH_2 did not form hydrogels within 24 h in Milli-Q water at 19 mg mL^{-1} and was therefore not included.]

2.3 Thixotropy

The recovery percentage was calculated based on the Equation S1:

$$\text{Recovey } \% = \frac{G'_{recov.}}{G'_{initial}} \times 100\% \quad \text{(S1)}$$

Table S1. Recovery percentage of P2 hydrogels with and without Zn(II) in 10% PBS.

Samples	G' recovered	G' initial	Recovery
P2 10% PBS	0.00456	0.00499	91.4%
$P2_{Zn}$ 10% PBS	0.04115	0.09521	43.2%

3. Bacteria growth inhibition zone assay

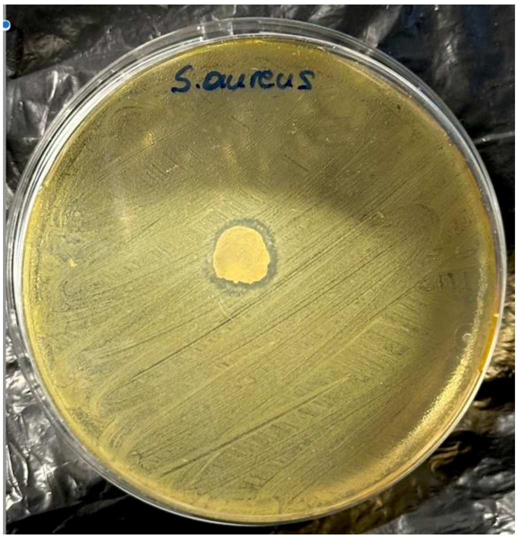

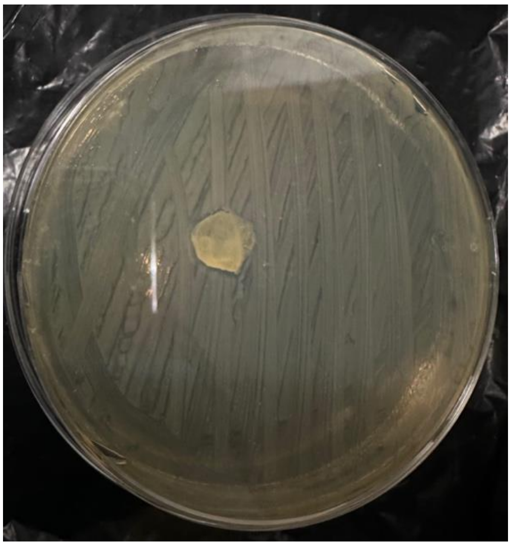

Figure S18: Agar diffusion assay showing antibacterial activity of Zn(II)-loaded peptide hydrogels. Representative images of agar plates after 24 hours of incubation at 37 °C. Left: *S. aureus*; Right: *E. coli*. Clear zones of inhibition surrounding the hydrogels indicate visible antibacterial activity against both Gram-positive and Gram-negative bacteria.
